# Optimized dose selective HDAC inhibitor tucidinostat overcomes anti-PD-L1 antibody resistance in experimental solid tumors

**DOI:** 10.1186/s12916-022-02598-5

**Published:** 2022-11-09

**Authors:** Pei Zhang, Yang Du, Hua Bai, Zhijie Wang, Jianchun Duan, Xin Wang, Jia Zhong, Rui Wan, Jiachen Xu, Xiran He, Di Wang, Kailun Fei, Ruofei Yu, Jie Tian, Jie Wang

**Affiliations:** 1 Department of Medical Oncology, National Cancer Center/National Clinical Research Center for Cancer/Cancer Hospital, Chinese Academy of Medical Sciences & Peking Union Medical College, Beijing, 100021 China; 2 CAS Key Laboratory of Molecular Imaging, Beijing Key Laboratory of Molecular Imaging, the State Key Laboratory of Management and Control for Complex Systems, Institute of Automation, Chinese Academy of Sciences, Beijing, 100190 China; 3 The University of Chinese Academy of Sciences, Beijing, 100049 China; 4 Department of Medical Oncology, Beijing Chest Hospital, Capital Medical University, Beijing Tuberculosis and Thoracic Tumor Research Institute, Beijing, 101125 China; 5 Beijing Advanced Innovation Center for Big Data-Based Precision Medicine, School of Medicine, Beihang University, Beijing, 100191 China; 6 School of Life Science and Technology, Xidian University, Xi’an, 710071 Shanxi China

**Keywords:** Tucidinostat, Tumor microenvironment, PD-L1, CCL5, Solid tumor

## Abstract

**Background:**

Although immune checkpoint inhibitors (ICIs) have influenced the treatment paradigm for multiple solid tumors, increasing evidence suggests that primary and adaptive resistance may limit the long-term efficacy of ICIs. New therapeutic strategies with other drug combinations are hence warranted to enhance the antitumor efficacy of ICIs. As a novel tumor suppressor, histone deacetylase (HDAC) inhibitor tucidinostat has been successfully confirmed to act against hematological malignancies. However, the underlying mechanisms of action for tucidinostat and whether it can manipulate the tumor microenvironment (TME) in solid tumors remain unclear.

**Methods:**

Three murine tumor models (4T1, LLC, and CT26) were developed to define the significant role of different doses of tucidinostat in TME. The immunotherapeutic effect of tucidinostat combined with anti-programmed cell death ligand 1 antibody (aPD-L1) was demonstrated. Furthermore, the effect of tucidinostat on phenotypic characteristics of peripheral blood mononuclear cells (PBMCs) from lung cancer patients was investigated.

**Results:**

With an optimized dose, tucidinostat could alter TME and promote the migration and infiltration of CD8^+^ T cells into tumors, partially by increasing the activity of C-C motif chemokine ligand 5 (CCL5) *via* NF-κB signaling. Moreover, tucidinostat significantly promoted M1 polarization of macrophages and increased the in vivo antitumor efficacy of aPD-L1. Tucidinostat also enhanced the expression of the costimulatory molecules on human monocytes, suggesting a novel and improved antigen-presenting function.

**Conclusions:**

A combination regimen of tucidinostat and aPD-L1 may work synergistically to reduce tumor burden in patients with cancer by enhancing the immune function and provided a promising treatment strategy to overcome ICI treatment resistance.

**Supplementary Information:**

The online version contains supplementary material available at 10.1186/s12916-022-02598-5.

## Background

Programmed cell death protein 1 (PD-1) is an immune checkpoint receptor expressed on activated T cells that modulate tissue immune tolerance [1, 2]. Tumor cells frequently overexpress the ligand programmed death ligand-1 (PD-L1), thereby facilitating their escape from immune surveillance [3–5]. Monoclonal antibodies (mAbs) against PD-1 or PD-L1 have demonstrated remarkable clinical efficacy in patients with a variety of cancers [3]. However, accumulating evidence suggests that mAbs against PD-1 and PD-L1 are less effective in non-inflamed tumors, indicating that such tumors are resistant to immune attack. Indeed, tumors unresponsive to PD-1 or PD-L1 mAbs are characterized by poor lymphocyte infiltration, low PD-L1 expression, and increased immunosuppressive factor expression in the tumor microenvironment (TME). Combining PD-1 or PD-L1 mAbs with certain agents that can modulate the immunosuppressive state may overcome the primary and adaptive resistance [6, 7]. Cytotoxic chemotherapy or molecularly targeted therapy has been demonstrated to enhance the effect of PD-1/PD-L1 mAbs as immunotherapeutic drugs [8–10].

Several studies have suggested a bidirectional relationship between epigenetic modifications and antitumor immunity in TME [11–13]. Cancers can be caused not only due to a change in the genomic DNA sequence but also through two typical epigenetic modifications: DNA methylation and histone modification [14]. These epigenetic modifications remodel the chromatin structure, thereby altering the gene expression profile and cell phenotype and potentially resulting in cell cycle dysregulation and tumor development [15–17]. Conversely, the reversal of histone and non-histone protein acetylation by histone deacetylase (HDAC) inhibitors can induce cell cycle arrest, differentiation, and cancer cell death [18, 19]. Such antitumor effects of HDAC inhibitors have been proven in human hematological tumors, but not yet in solid tumors. Recently, preclinical studies have reported the efficacy of HDAC inhibitors combined with other therapeutic agents, including chemotherapy or targeted drugs, in treating solid tumors [20–22]. Trials investigating these combinations in patients with solid tumors are also ongoing, and the preliminary data obtained seem promising. However, the antitumor effects of HDAC inhibitors combined with immune checkpoint inhibitors (ICIs) have not been extensively studied [23, 24]. Challenges remain in harnessing the full potential of combined HDAC inhibition and immunotherapy and selecting optimal regimens for different solid tumors.

Tucidinostat, an oral HDAC inhibitor belonging to the benzamide class and having specificity for HDAC1, HDAC2, HDAC3, and HDAC10 subtypes, has been approved for the treatment of relapsed or refractory peripheral T cell lymphoma and is under clinical development globally for various other neoplastic and non-neoplastic diseases [25–29]. A recent phase III trial reported that tucidinostat combined with endocrine blockade could be effective against advanced hormone receptor-positive HER2-negative breast cancer progression after endocrine therapy alone [30]. Additionally, grade 3 or 4 toxicities including neutropenia, leucopenia, and thrombocytopenia were more frequent in the tucidinostat group than in the placebo group, indicating that tucidinostat may induce immunosuppression, which is not conducive to effective immunotherapy. Therefore, assessing the effect of different doses of tucidinostat in the presence or absence of ICIs on solid tumor growth and TME immune status is warranted to discover an optimized combination therapy.

Here, we analyzed the antitumor efficacy of different doses of tucidinostat alone and in combination with aPD-L1 in three murine solid tumor models that were unresponsive or transiently responsive to ICIs to explore the optimal strategy for combining tucidinostat and aPD-L1, as well as its underlying mechanisms. This may provide guidance to improve the clinical management of combined immunotherapy.

## Methods

### Materials and reagents

Tucidinostat (Cat# HY-109015), vorinostat (Cat# HY-10221), TMP-195 (Cat# HY-18361), and NF-κB inhibitor BAY11-7082 (Cat# HY-13453) were obtained from MCE (Monmouth Junction, NJ, USA). Dimethyl sulfoxide (DMSO) has been used as vehicle control for each drug.

### Cell culture

4T1 breast cancer cells, Lewis lung cancer (LLC) cells, CT26 colorectal cancer cells, and Raw 264.7 cells were purchased from the Chinese Academy of Sciences (Beijing, China) and cultured in RPMI-1640 medium (Hyclone) containing 10% fetal bovine serum at 37°C in a 5% CO_2_ incubator.

### Establishment of murine syngeneic tumor models

For subcutaneous injections, mouse 4T1, LLC, and CT26 cells (5 × 10^5^ cells) were injected into the right flank of BALB/c or C57 BL/6 mice. When established tumors were palpable 7 days after tumor cell inoculation, mice were treated with different doses of tucidinostat (MCE, Cat# HY-109015, 12.5, 25, 75 mg/kg, oral gavage, daily) and aPD-L1 (BioXcell, Cat# BE0101; 200 μg, intraperitoneal injection, every 3 days). The volume of tumor nodules was measured every 3 days and calculated as V = (a × b^2^)/2, where “a” and “b” are the long and short axis of the tumor nodule, respectively. Mice were monitored until their individual tumor volume reaches the approved protocol volume limit (2000mm^3^). At the treatment, the tumor-bearing mice were anesthetized and tissues were harvested for further analysis.

Depletion of CD8^+^ T cells was performed by intraperitoneal injection of anti-mouse CD8a (aCD8, BioXcell, Cat# BP0117; 200 μg, every 3 days). After aCD8 treatment, the percentage of CD8^+^ T cells (CD3^+^CD4^−^CD8^+^ T cells) was significantly decreased in the tumor and spleen tissues.

Depletion of macrophage was performed by intraperitoneal injection of clodronate liposomes (FormuMax, Cat# F70101C-A; 1.4 mg/20g body weight, every 3 days), respectively. After clodronate liposome treatment, the percentage of macrophages (CD11b^+^F4/80^+^ macrophages) was significantly decreased in the tumor and spleen tissues.

Animal studies were conducted in accordance with the NIH animal use guidelines and approved by the Institutional Review Board of the National Cancer Center/National Clinical Research Center for Cancer/Cancer Hospital, Chinese Academy of Medical Sciences & Peking Union Medical College (Permit Number, NCC2020A167).

### Quantitative real-time PCR (RT-qPCR)

Total RNAs were extracted using the RNeasy Kit (Takara Bio). The qRT-PCR was carried out using SYBR Green Premix Ex TaqTM II (Takara Bio) on a ABI StepOnePlus Real-Time PCR Detection System (Thermo Fisher Scientific). Results were normalized to the housekeeping gene GAPDH. Relative gene expression levels from different groups were calculated with the 2-ΔΔCT method and compared with the expression level of appropriate control cells.

Specific primer sequences for individual genes were as follows: CCL5 (forward: 5′-GTATTTCTACACCAGCAGCAAG-3′; reverse: 5′-TCTTGAACCCACTTCTTCTCTG-3′); CXCL9 (forward: 5′-AATCCCTCAAAGACCTCAAACA-3′; reverse: 5′-TCCCATTCTTTCATCAGCTTCT-3′); CXCL10 (forward: 5′-CAACTGCATCCATATCGATGAC-3′; reverse: 5′-GATTCCGGATTCAGACATCTCT-3′); PD-L1 (forward: 5′-TGAGCAAGTGATTCAGTTTGTG-3′; reverse: 5′-CATTTCCCTTCAAAAGCTGGTC-3′); iNOS (forward: 5′-GCCGAGTGCAAGCATGGAGAG-3′; reverse: 5′-GGCTGTGAGGTGAGGTTGAAGAAG-3′); CD86 (forward: 5′-ACGGAGTCAATGAAGATTTCCT-3′; reverse: 5′-GATTCGGCTTCTTGTGACATAC-3′); CD206 (forward: 5′-CCTATGAAAATTGGGCTTACGG-3′; reverse: 5′-CTGACAAATCCAGTTGTTGAGG-3′); Arg1 (forward: 5′-CATATCTGCCAAAGACATCGTG-3′; reverse: 5′-GACATCAAAGCTCAGGTGAATC-3′); and GAPDH (forward: 5′-GTATTTCTACACCAGCAGCAAG-3′; reverse: 5′-TCTTGAACCCACTTCTTCTCTG-3′).

### Western blot analysis

The cell culture dish was placed on ice and add with ice-cold lysis buffer. The cell suspension was gently transferred into a pre-cooled microcentrifuge tube and centrifuged in a microcentrifuge at 4°C for 20 min at 12,000 rpm. The protein was collected and separated by 10% SDS-PAGE gel and transferred onto a polyvinylidene difluoride membrane (Millipore). Subsequently, the membrane was blocked and incubated overnight at 4°C with the primary antibody including anti-CCL5 mAb (1: 500, Abcam, Cat# ab7198) and anti-phospho-NF-κB p65 (1:1000, Cell Signaling Technology, Cat# 3039). The same membrane was probed for GAPDH (1:10,000, Abcam, Cat# ab8245) as the internal control. After washing with TBST solution, the membrane was incubated with the corresponding secondary Abs. Finally, the blots were developed in ECL reagent (Thermo Fisher Scientific Inc, Cat# 32209) and imaged using the ImageQuant LAS 500 system (GE Healthcare).

### CCK-8 assay

To assess the effect of different doses of tucidinostat on cell proliferation, 4T1, LLC, and CT26 cells were plated in 96-well plates at a started number of 3 × 10^3^ cells/well and treated with different doses (2.5, 5, 7.5 μM) of tucidinostat for 24 h. The absorbance of each sample was measured using a Cell Counting Kit-8 (CCK-8) kit (Solarbio) on a microplate reader (Thermo Scientific) at 450 nm. The ratio of the optical density (OD) value of each cell group normalized to the cells without tucidinostat treatment is presented. The experiment was performed in triplicate.

### Annexin V-FITC/PI assay

To assess the effect of different doses of tucidinostat on cell apoptosis, 4T1, LLC, and CT26 cells were plated in 6-well plates at a started number of 3 × 10^5^ cells/well and treated with different doses (2.5, 5, 7.5 μM) of tucidinostat for 6 h and then were collected and incubated with Annexin V-FITC and PI (Beyotime Biotechnology). After a 15-min incubation period at room temperature, the cells were analyzed by flow cytometric analysis (CytoFLEX, Beckman). Data was analyzed using the FlowJo software (Ashland, OR, USA). The experiment was performed in triplicate.

### Flow cytometric analysis

Tumors from the subcutaneous tumor model were harvested for single-cell suspensions using a tumor dissociation kit (Miltenyi Biotec GmbH). The drainage lymph nodes (dLNs) were harvested through mechanical dissociation. Dissociated cells were filtered through a 4μm strainer and suspended in phosphate-buffered saline (PBS) supplemented with 1% FBS. The cells were stained with the following Abs according to the manufacturer’s instructions: CD45 (Cat# 103114), CD3 (Cat# 100204), CD4 (Cat# 100414), CD8a (Cat# 100752), CD25 (Cat# 101923), CD44 (Cat# 103031), CD62L (Cat# 161203), F4/80 (Cat# 123128), CD11b (Cat# 101245), MHC-II (Cat# 107606), CD206 (Cat# 141708), Gr-1 (Cat# 108423), CD11c (Cat# 117329), CD86 (Cat# 105014), PD-1 (Cat# 135206), or PD-L1 (Cat# 124312) (2.5μl, all from BioLegend) were diluted in FACS buffer (Biolegend).

Various immune cells were separated using a gating strategy based on the expression of known lineage markers for lymphocytes (CD45^+^), total T cells (CD45^+^CD3^+^), CD4^+^ T cells (CD45^+^CD3^+^CD4^+^), CD8^+^ T cells (CD45^+^CD3^+^CD8^+^), Treg cells (CD45^+^CD3^+^CD4^+^CD25^+^), central memory T cells (CD3^+^CD4^+^CD44^+^CD62L^+^), effective memory T cells (CD3^+^CD4^+^CD44^+^CD62L^−^), macrophages (CD45^+^CD11b^+^F4/80^+^), M1 macrophages (CD45^+^CD11b^+^F4/80^+^/MHC-II^+^), DC cells (CD45^+^CD11b^−^CD11c^+^), MDSCs (CD45^+^CD11b^+^Gr-1^+^), and NK cells (CD45^+^CD3^−^CD49b^+^).

Data was performed on the flow cytometers (Cytek NL-CLC 3000, Cytek) and analyzed using the FlowJo software (Ashland, OR, USA).

### Immunohistochemistry (IHC)

Tumors were collected and fixed in 4% formalin. Sections of paraffin-embedded tissues (4 μm) were deparaffinized in xylene and rehydrated in a graded series of alcohol concentrations. 3% H_2_O_2_ was used to block endogenous peroxidase activity, and the slides were incubated in Tris-EDTA buffer for antigen retrieval. Subsequently, the sections were incubated overnight at 4°C with the primary antibody against CD3 (Cat# ab16669), CD4 (Cat# ab183685), or CD8 (Cat# ab217344) (1:200, all from Abcam) overnight. Sections incubated with normal mouse or rabbit IgG instead of primary antibodies were used as the negative control. For IHC, the sections were incubated with the HRP-linked secondary Ab and the cell nucleus was counterstained using hematoxylin. For immunofluorescence staining, the sections were incubated with the fluorophore-conjugated secondary Abs and the cell nucleus was counterstained using 4′,6-diamidino-2-phenylindole (DAPI).

### Genomic analysis

Mouse CT26 cells (5 × 10^5^ cells) were engrafted into the flank of BALB/c mice. When established tumors were palpable 7 days after tumor cell inoculation, mice were treated with tucidinostat (25 mg/kg, gavage, daily, *n*=3) or DMSO as vehicle control (DMSO, *n*=3). To investigate the intrinsic mechanisms of tucidinostat on tumor immune microenvironment, tumor tissue from CT26 tumor-bearing mice on day 10 post-treatment initiation was harvested using TRIzol (Invitrogen) according to manufacturer’s instructions (Novogene co., Ltd). Total RNA was used as input material for the RNA sample preparations. PCR products were purified (AMPure XP system) and library quality was assessed on the Agilent Bioanalyzer 2100 system. After cluster generation, the library preparations were sequenced on an Illumina Novaseq platform and 150-bp paired-end reads were generated. Differential expression analysis of two groups was performed using the DESeq2 R package (1.20.0). Gene Ontology (GO) enrichment analysis of differentially expressed genes was implemented by the cluster Profiler R package, in which gene length bias was corrected. For tumor environment analysis, published mRNA signatures for T cells and other cell clusters were analyzed [31, 32].

### Mouse peripheral blood preparation and cytokine assay

To assess the effect of different doses of tucidinostat on hepatic/renal toxicity and cytokine secretion, peripheral blood was collected from the inner canthus of the experimental mice. Mouse white blood cell (WBC) count, red blood cell (RBC) count, platelet (PLT) count, and lymphocyte count were detected by a fully automatic hematology analyzer (BC-2800 Vet, Mindray).

Peripheral blood was collected and then centrifuged at 3000 rpm for 10 min to isolate the serum. The serum alanine transaminase (ALT), alanine transaminase (ALT), and urea nitrogen (BUN) (all from Anoric Biotechnology) were measured using ELISA kits. The serum IL-10 (Cat# 431417), IFN-γ (Cat# 430807), TNF-α (Cat# 430907) (all from Biolegend), and CCL5 (R&D, Cat# DY478) were measured using ELISA kits. The absorbance of each sample was measured on a microplate reader (Thermo Scientific) at 450 nm.

### Ex vivo chemotaxis assay

To further confirm the chemotactic effect of CCL5 on CD8^+^ T cells, naïve CD8^+^ T cells were purified from mouse spleen and activated with Dynabeads containing mouse T-activator CD3 (Biolegend, Cat# 100301)/CD28 (Biolegend, Cat# 102101) and recombinant mouse IL-2 (Biolegend, Cat# 714604). And then, the activated CD8^+^ T cells were seeded in the upper chambers of transwell plates (BD Biosciences) and allowed to migrate for 24 h towards the lower chamber containing medium with different concentrations of CCL5 protein (Pepro Tech, Cat# 250-07).

### BMDM preparation

Bone marrow cells were isolated from femurs and tibias of C57/BL6 mice. 5 × 10^6^ cells per well in 24-well plates were cultured in RPMI-1640 medium containing 10% heated-inactivated fetal bovine serum at 37°C in a 5% CO_2_ incubator. Bone marrow-derived macrophages (BMDMs) were differentiated in the presence of recombinant cytokine M-CSF (20ng/ml, Pepro Tech, Cat# 315-02). Every 2 days, 50% of the medium were replaced with fresh culture medium. After 10 days, we harvested adherent cells and used them for BMDM experiments.

### PBMC preparation

To determine the effect of different doses of tucidinostat in human leukocytes, the peripheral blood mononuclear cells (PBMCs) were isolated from fresh blood samples obtained from non-small cell lung cancer (NSCLC) patients and rested in RPMI-1640 medium containing 10% heated-inactivated fetal bovine serum at 37°C in a 5% CO_2_ incubator for 6 h. Then, 5 × 10^5^ cells per well in 24-well plates were cultured with different doses of tucidinostat as indicated for 24 h.

The cells were stained with the following Abs according to the manufacturer’s instructions: CD14 (Cat# 325604), CD11b (Cat# 393112), CD3 (Cat# 344804), CD4 (Cat# 317428), CD8a (Cat# 344722), CD69 (Cat# 310906), CD86 (Cat# 374208), or HLA-DR (Cat# 307630) (2.5μl, all from BioLegend) were diluted in FACS buffer (Biolegend).

Various immune cells were separated using a gating strategy based on the expression of known lineage markers for total peripheral blood monocytes (CD14^+^CD11b^+^), active peripheral blood monocytes (CD14^+^CD11b^+^HLA-DR^+^/CD14^+^CD11b^+^CD86^+^), total T cells (CD3^+^), CD4^+^ T cells (CD3^+^CD4^+^), CD8^+^ T cells (CD3^+^CD8^+^), active CD4^+^ T cells (CD3^+^CD4^+^CD69^+^), and active CD8^+^ T cells (CD3^+^CD8^+^CD69^+^). Data was collected and analyzed with the flow cytometers (BD Accuri® C6, BD).

The study was approved by the Institutional Review Board of the National Cancer Center/National Clinical Research Center for Cancer/Cancer Hospital, Chinese Academy of Medical Sciences & Peking Union Medical College (Permit Number, NCC2020C072).

### Statistical analysis

All data analysis was performed using GraphPad Prism software (version 5.0, GraphPad Software, Inc.). For the comparison among treatment groups in the in vitro and in vivo study, one-way ANOVA was performed. Survival time was defined from the day of tumor cell inoculation until the mice expired naturally or were euthanized. Survival curves were drawn using the Kaplan-Meier method and compared with the log-rank test. *P*<0.05 was considered statistically significant. In the figures, symbols were used as *P<0.05, **P<0.01, and ***P<0.001.

## Results

### Optimized dose of tucidinostat inhibits tumor growth and activates the tumor immune microenvironment

**Fig. 1:**
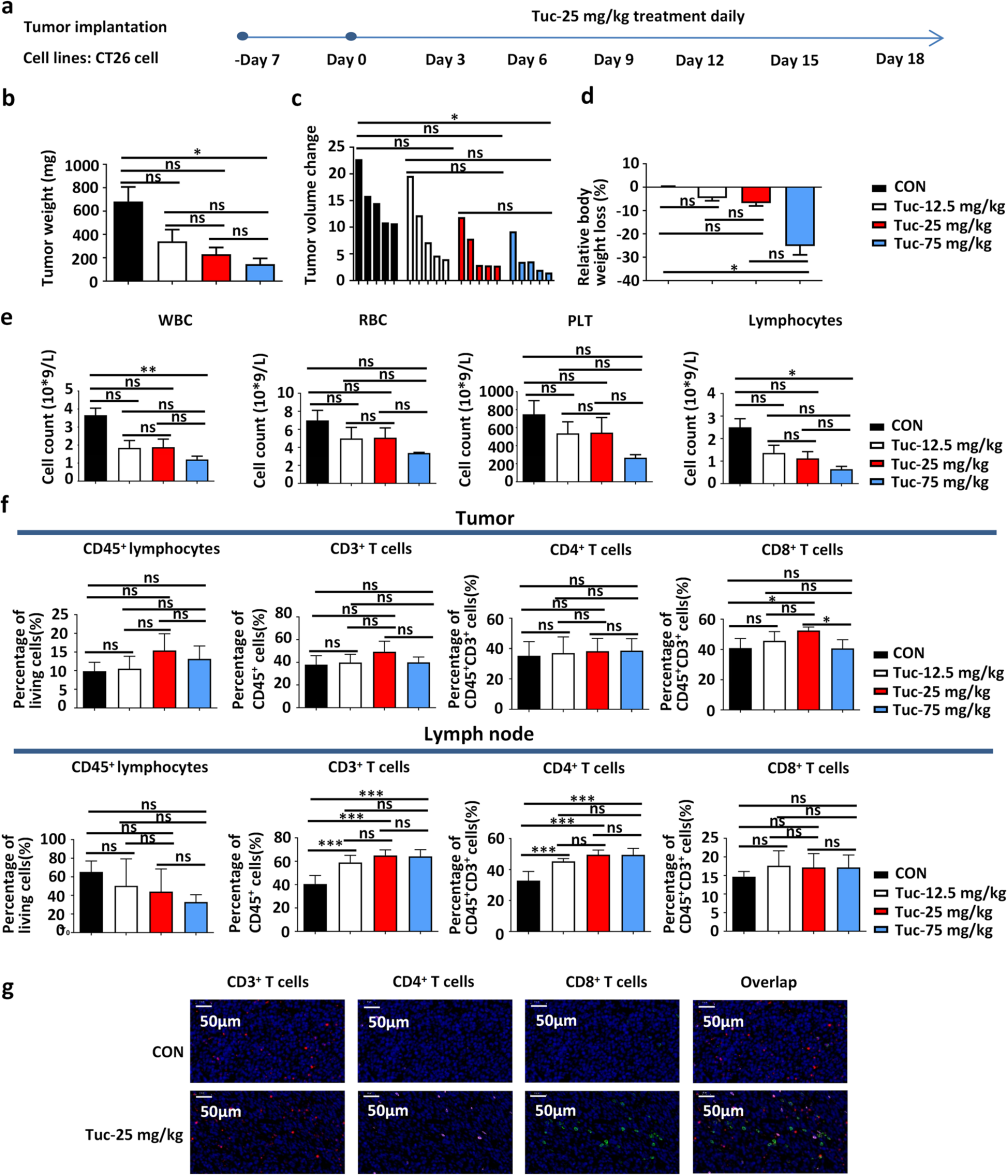
Optimized dose tucidinostat inhibits tumor growth via modulating the antitumor immune response. **a** Schema of the experiment. Mouse CT26 cells (5 × 10^5^ cells) were engrafted into the flank of BALB/c mice. When established tumors were palpable 7 days after tumor cell inoculation, mice were treated with different doses (12.5, 25, 75 mg/kg, gavage, daily, *n*=5) of tucidinostat or DMSO as vehicle control (DMSO, *n*=5). **b** Tumor weight on day 10 post-treatment initiation. **c** Waterfall plot of individual tumor volume changes on day 10 post-treatment initiation. The individual tumor volume change means the ratio of the tumor volume on day 10 to that on day 0 after tucidinostat treatment. **d** The percentage of body weight change on day 10 post-treatment initiation. **e** Hematological parameters on day 10 post-treatment initiation. The blood samples were collected and determined using a routine blood test. Routine blood tests include WBC count, RBC count, PLT count, and lymphocyte count. **f** Flow cytometric quantification of lymphocytes (CD45^+^), total T cells (CD45^+^CD3^+^), CD4^+^ T cells (CD45^+^CD3^+^CD4^+^), and CD8^+^ T cells (CD45^+^CD3^+^CD8^+^) in tumor parenchyma and tumor drainage lymph nodes from CT26 tumor-bearing mice on day 10 post-treatment initiation. **g** Representative immunofluorescent staining for tumor-infiltrating T cells on day 10 post-treatment initiation. Red, CD3 staining; pink, CD4 staining; green, CD8 staining; blue, DAPI staining. The error bars indicate mean ± SEM. *P<0.05, **P<0.01, ***P<0.001 by one-way ANOVA. ns not significant, WBC white blood cell, RBC red blood cell, PLT platelet, CON control group, Tuc tucidinostat

The antitumor effect of different doses (2.5, 5, 7.5 μM) of tucidinostat was first evaluated in three different cell lines—4T1 breast cancer cells, LLC lung cancer cells, and CT26 colorectal cancer cells—in vitro and the data revealed that the cell proliferation levels were significantly suppressed and the cell apoptosis levels were increased in the higher-dose groups (Additional file 1: Fig. S1a-b). To determine the optimized dose of tucidinostat, the in vivo activity of tucidinostat was assessed in the CT26 tumor-bearing mice. The mice were gavaged with tucidinostat daily at three different doses (12.5, 25, and 75 mg/kg) after tumor cell inoculation (Fig. 1a). The administration of the lower dose (12.5 mg/kg) and middle dose (25 mg/kg) of tucidinostat induced sustained and modest decrease of tumor growth with tolerable toxicity, and the higher dose (75 mg/kg) of tucidinostat induced significantly greater tumor growth suppression but with intolerable toxicities, such as rapid body weight loss, leucopenia, and lymphopenia (Fig. 1b–e). The higher dose (75 mg/kg) of tucidinostat also elevated the serum alanine transaminase (ALT) level, which indicated the impaired liver function. To evaluate kidney function, blood urea nitrogen (BUN) levels were measured; less damage was recorded in these groups (Additional file 1: Fig. S1c).

Immunological changes occurring in TME after tucidinostat treatment were further assessed. Flow cytometry data demonstrated a significant increase in the number of CD8^+^ T cells infiltrating tumors at 10 days post-administration in the tucidinostat (25 mg/kg) group, indicating that this dose exerted a robust immune priming effect (Fig. 1f). Moreover, the CD8^+^ T cells in the drainage lymph nodes (dLNs) also increased, although the difference was not statistically significant. Interestingly, the number of CD3^+^ and CD4^+^ T cells increased following tucidinostat (25 mg/kg) treatment, suggesting that the treatment induced effective antitumor immune responses (Fig. 1f). Furthermore, immunofluorescence staining of tumor sections demonstrated that the proportion of CD8^+^ T cells was higher in this group (Fig. 1g). Considering the antitumor efficacy and safety profile, the optimized dose (25 mg/kg) of tucidinostat was selected for further investigations.

### Tucidinostat induces the expression of effector T cell-attracting chemokines and PD-L1

**Fig. 2:**
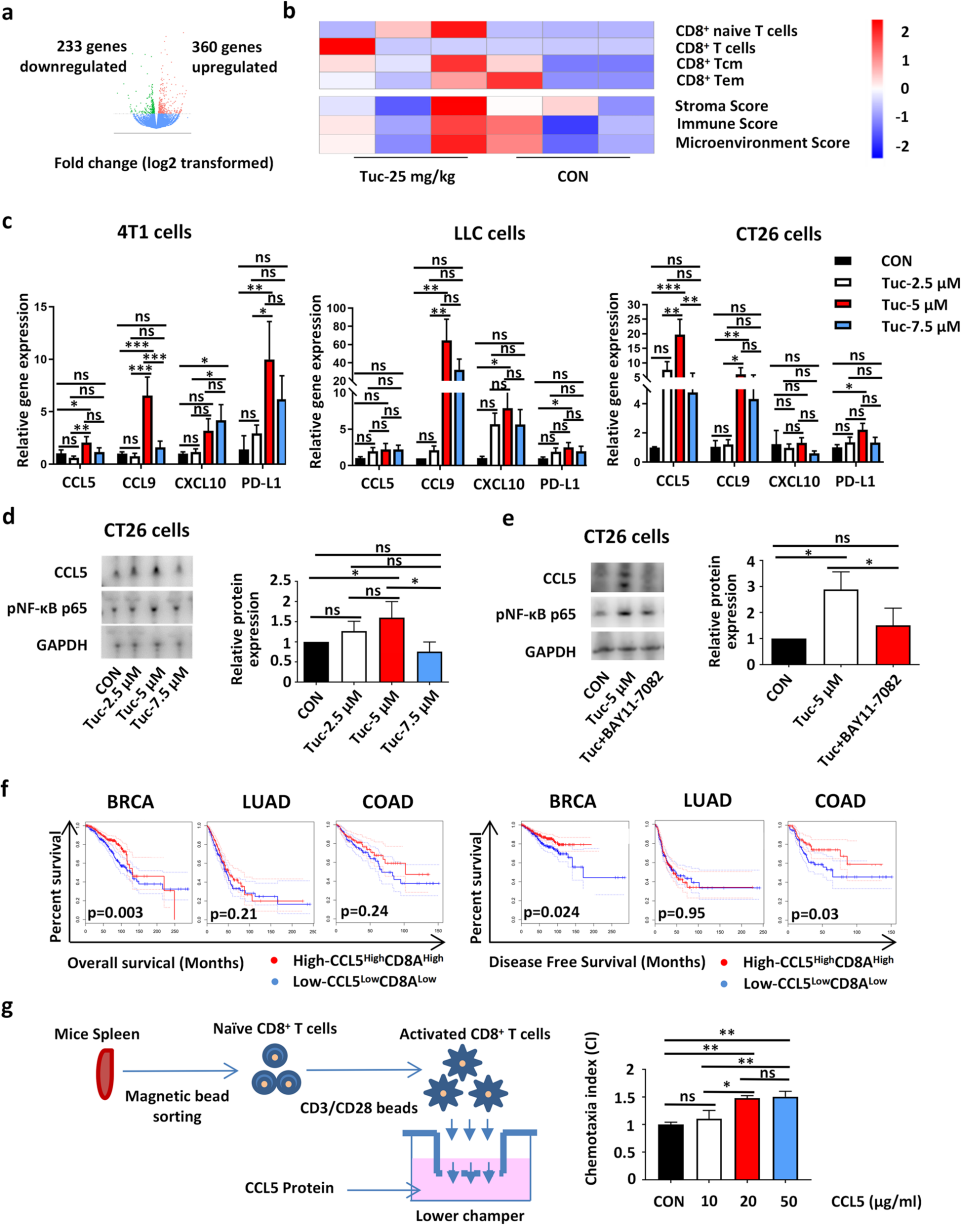
Optimized dose tucidinostat promotes CD8^+^ T cell migration by inducing pro-inflammatory CCL5 secretion in tumor. **a** Volcano plot showing the significantly overexpressed genes (red) and significant underexpressed genes (green) in tumor from CT26 tumor-bearing mice on day 10 post-treatment with tucidinostat (25 mg/kg, gavage, daily, *n*=3) or DMSO as vehicle control (DMSO, *n*=3). **b** Heatmap showing the scores of immune gene signatures in tumor from CT26 tumor-bearing mice on day 10 post-treatment initiation. Colors in the heatmap represent the level of significance of the enrichment (−log10 of the adjusted *p* values). **c** Relative mRNA expression of CCL5, CXCL9, CXCL10, and PD-L1 compared to vehicle (set to fold change = 1) in 4T1, LLC, and CT26 cells that were exposed to increasing concentrations (2.5, 5, 7.5 μM) of tucidinostat for 24 h. The experiment was performed in triplicate. **d** Relative protein expression of CCL5 and phospho-p65-NF-κB (pNF-κB p65) compared to vehicle in CT26 cells that were exposed to increasing concentrations (2.5, 5, 7.5 μM) of tucidinostat for 48 h. The experiment was performed in triplicate. **e** Relative protein expression of CCL5 and pNF-κB p65 compared to vehicle in CT26 cells that were exposed to tucidinostat and NF-κB inhibitor (BAY11-7082) for 48 h. The experiment was performed in triplicate. **f** Kaplan-Meier survival curves for overall survival and disease-free survival in three solid tumor types as stratified by CCL5^High^CD8A^High^ or CCL5^low^CD8A^low^ expression status using the TCGA database. **g** CD8^+^ T cell migration with different doses of CCL5 protein (10, 20, 50μg/ml) for 24h. The experiment was performed in triplicate. The error bars indicate mean ± SEM. *P<0.05, **P<0.01, ***P<0.001 by one-way ANOVA. ns not significant, BRCA breast invasive carcinoma, LUAD lung adenocarcinoma, COAD colon adenocarcinoma, CON control group, Tuc tucidinostat, phospho-p65-NF-κB pNF-κB p65

Next, potential mechanisms underlying the changes in TME were explored through gene expression profiling. Bulk mRNA-seq data comparing tucidinostat-treated (25 mg/kg) and untreated tumors from the CT26 murine model indicated that tucidinostat could markedly alter the TME, as indicated by the significantly higher immune microenvironment scores. A heatmap of differential expressed genes revealed a consistency between immune-related gene signatures and the changes observed in the T cell populations (Fig. 2a, b). Furthermore, the functional annotation of gene clustering indicated altered expression levels of a considerable amount of cytokines following tucidinostat treatment (Additional file 2: Fig. S2a).

It is known that the best predictors of immunotherapy response are the number and phenotype of tumor-infiltrating CD8^+^ T cells recruited at the tumor site by the locally secreted chemokines [33, 34]. A large body of evidence exists to show that increased expression of CD8^+^ T cell-attracting chemokines, such as the C-C motif chemokine ligand 5 (CCL5) and C-X-C motif chemokine ligand 9 and 10 (CXCL9 and CXCL10), correlates with decreased levels of cancer metastasis and improved clinical outcome in patients with cancer [35–37]. Recently, a mechanistic link between epigenetic modification and the secretion of such cytokines in TME has been described [38, 39]. Here, we hypothesized that the immune modulation of tucidinostat may occur, at least partially, through tumor-derived cytokine secretion. qPCR was performed for the treated murine 4T1, LLC, and CT26 cancer cell lines, and the data showed that the total expression levels of CCL5, CXCL9, and CXCL10 were significantly increased after tucidinostat treatment (Fig. 2c). More importantly, the optimized dose of tucidinostat treatment also improved the total and cell surface expressions of PD-L1 (Fig. 2c, Additional file 2: Fig. S2b). In general, tucidinostat at the optimized dose could elevate the expression of PD-L1 and T cell-attracting chemokines, such as CCL5, CXCL9, and CXCL10.

Considering that the stress-activated NF-κB pathway controls cytokine expression in multiple cell types and the key role of CCL5 in attracting CD8^+^ T cells [40, 41], we further hypothesized that the antitumor immune response induced by tucidinostat may be mediated through CCL5 upregulation via the NF-κB pathway. As expected, NF-κB pathway was activated along with an increased expression of CCL5 after the administration of tucidinostat treatment in CT26 cancer cell lines, and this upregulation of CCL5 expression was abrogated upon pharmacological NF-κB inhibition using BAY11-7082 (Fig. 2d, e).

Previous data suggested that high intratumoral expression of CD8^+^ T cell-attracting chemokine CCL5 is correlated with better prognosis in several types of cancers [42, 43]. Moreover, CD8A expression is significantly correlated with CD8^+^ T cell infiltration and surface proteins that are critical to transduce antigen recognition into immune cell responses [44]. Therefore, the prognostic roles of CCL5 and CD8A were evaluated using public datasets from The Cancer Genome Atlas (TCGA). The analysis revealed that CCL5 expression is positively correlated with CD8A expression in three cancer types (breast invasive carcinoma, lung adenocarcinoma, and colon adenocarcinoma) (Additional file 2: Fig. S2c). Furthermore, both CCL5^High^ and CCL5^High^ CD8A^High^ are negatively correlated with the risk of death or recurrence in breast cancer (Fig. 2f, Additional file 2: Fig. S2d), which is consistent with previous data. Ex vivo chemotaxis assays have also shown that higher CCL5 protein concentrations could enhance CD8^+^ T cell transwell migration (Fig. 2g), suggesting that CCL5 is necessary for T cell infiltration.

Overall, these findings demonstrated a strong intrinsic anticancer activity of tucidinostat, which was mediated by the enhancement of CD8^+^ T cell recruitment through CCL5 upregulation via NF-κB signaling pathway activation.

### Tucidinostat enhances M1 polarization of macrophages both in vitro and in vivo

**Fig. 3:**
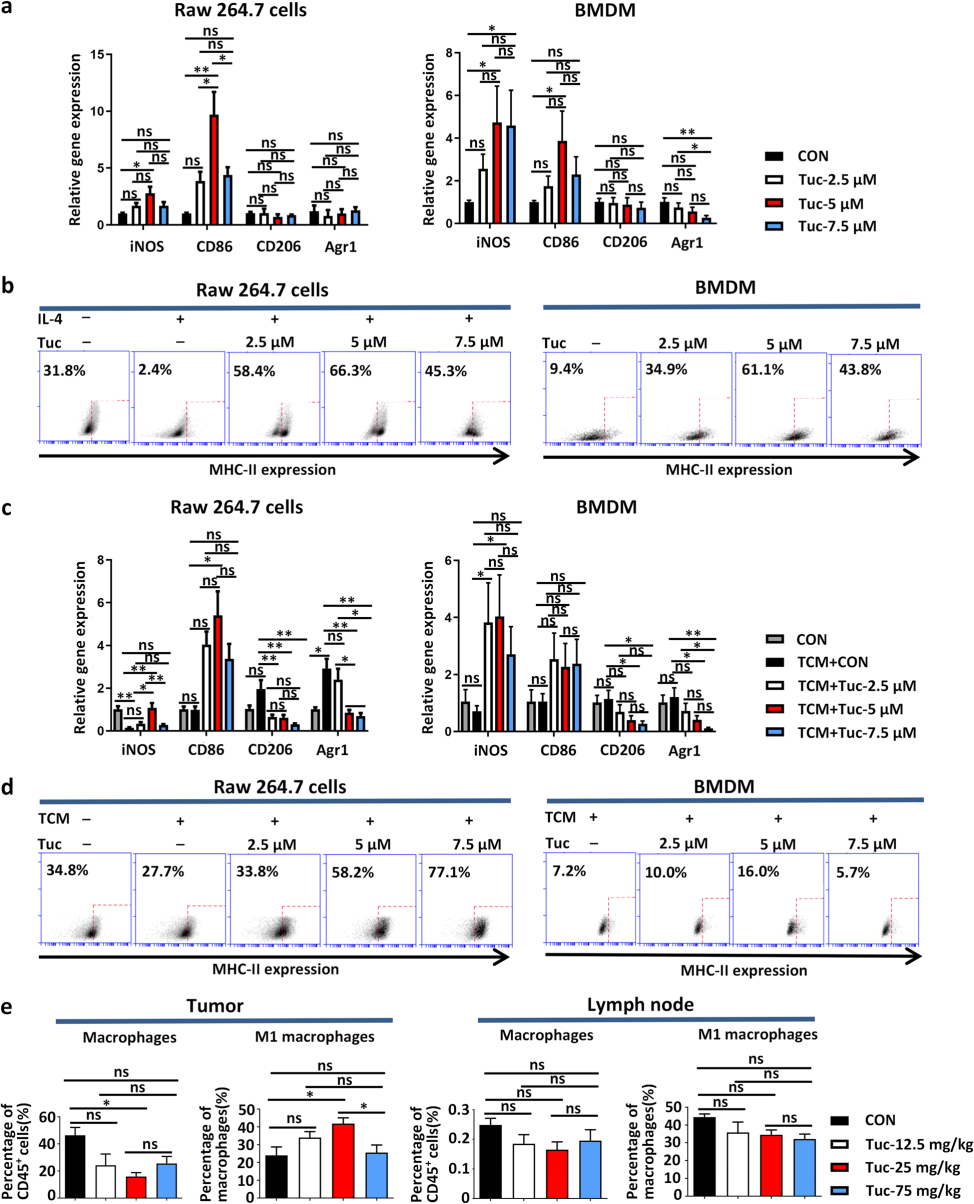
Tucidinostat enhances macrophage M1 polarization in vitro and in vivo. **a** Relative mRNA expression of iNOS, CD86, CD206, and Arg 1 compared to vehicle (set to fold change=1) in Raw.264.7 cells and BMDMs that were exposed to increasing concentrations (2.5, 5, 7.5 μM) of tucidinostat for 24 h. The experiment was performed in triplicate. **b** Representative cytograms for the expression levels of MHC-II on Raw.264.7 cells and BMDMs that were exposed to increasing concentrations (2.5, 5, 7.5 μM) of tucidinostat for 24 h. **c** LLC cells being exposed to increasing concentrations (2.5, 5, 7.5 μM) of tucidinostat for 24 h and the tumor-conditioned medium being collected and added into Raw.264.7 cells and BMDMs for 24 h. Relative mRNA expression of iNOS, CD86, CD206, and Arg 1 compared to vehicle (set to fold change=1) in Raw.264.7 cells and BMDMs that were exposed to such tumor-conditioned medium. The experiment was performed in triplicate. **d** Representative cytograms for the expression levels of MHC-II on Raw.264.7 cells and BMDMs that were exposed to such tumor-conditioned medium. **e** Mouse CT26 cells (5 × 10^5^ cells) were engrafted into the flank of BALB/c mice. When established tumors were palpable 7 days after tumor cell inoculation, mice were treated with different doses (12.5, 25, 75 mg/kg, gavage, daily, *n*=5) of tucidinostat or DMSO as vehicle control (DMSO, *n*=5). Percentage of total macrophages (CD45^+^CD11b^+^F4/80^+^) and the percentage of M1 macrophages (CD45^+^CD11b^+^F4/80^+^/MHC-II^+^) in tumor parenchyma and tumor drainage lymph nodes from CT26 tumor-bearing mice on day 10 post-treatment initiation. The error bars indicate mean ± SEM. *P<0.05, **P<0.01, ***P<0.001 by one-way ANOVA. ns not significant, BMDM bone marrow-derived macrophage, CON control group, TCM tumor-conditioned medium, Tuc tucidinostat, iNOS inducible nitric oxide synthase, Arg 1 arginase-1

Next, we examined whether tucidinostat could induce an innate antitumor immune response that leads to effective tumor regression. Several immune-related gene expressions were observed in Raw 264.7 cells and bone marrow-derived macrophages (BMDMs) 24h after the administration of tucidinostat treatment. mRNA expression levels of the M1-like macrophage marker inducible nitric oxide synthase (iNOS) and CD86 were increased (Fig. 3a). Furthermore, flow cytometry analysis revealed dose-dependent elevations of M1-like macrophage surface marker, MHC-II, on both Raw.264.7 cells and BMDMs (Fig. 3b). The tumor-conditioned medium collected from LLC cells after tucidinostat treatment also promoted M1 polarization of Raw.264.7 cells and BMDMs as assessed by qPCR and flow cytometry (Fig. 3c, d).

To further investigate the influence of tucidinostat on macrophage polarization in vivo, the immunological changes observed in tumors after administering treatment to CT26 tumor-bearing mice were assessed. Compared with other groups, a significant decrease in the proportion of tumor-associated macrophages among the total viable cells and an increase in the ratio of M1 macrophage were observed in the tucidinostat-treated (25 mg/kg) group (Fig. 3e). Total macrophages in dLNs also distinctly decreased, although the downward trend did not reach statistical difference (Fig. 3e).

### Tucidinostat improves the immunotherapeutic efficacy of ICI and induces a durable response

**Fig. 4:**
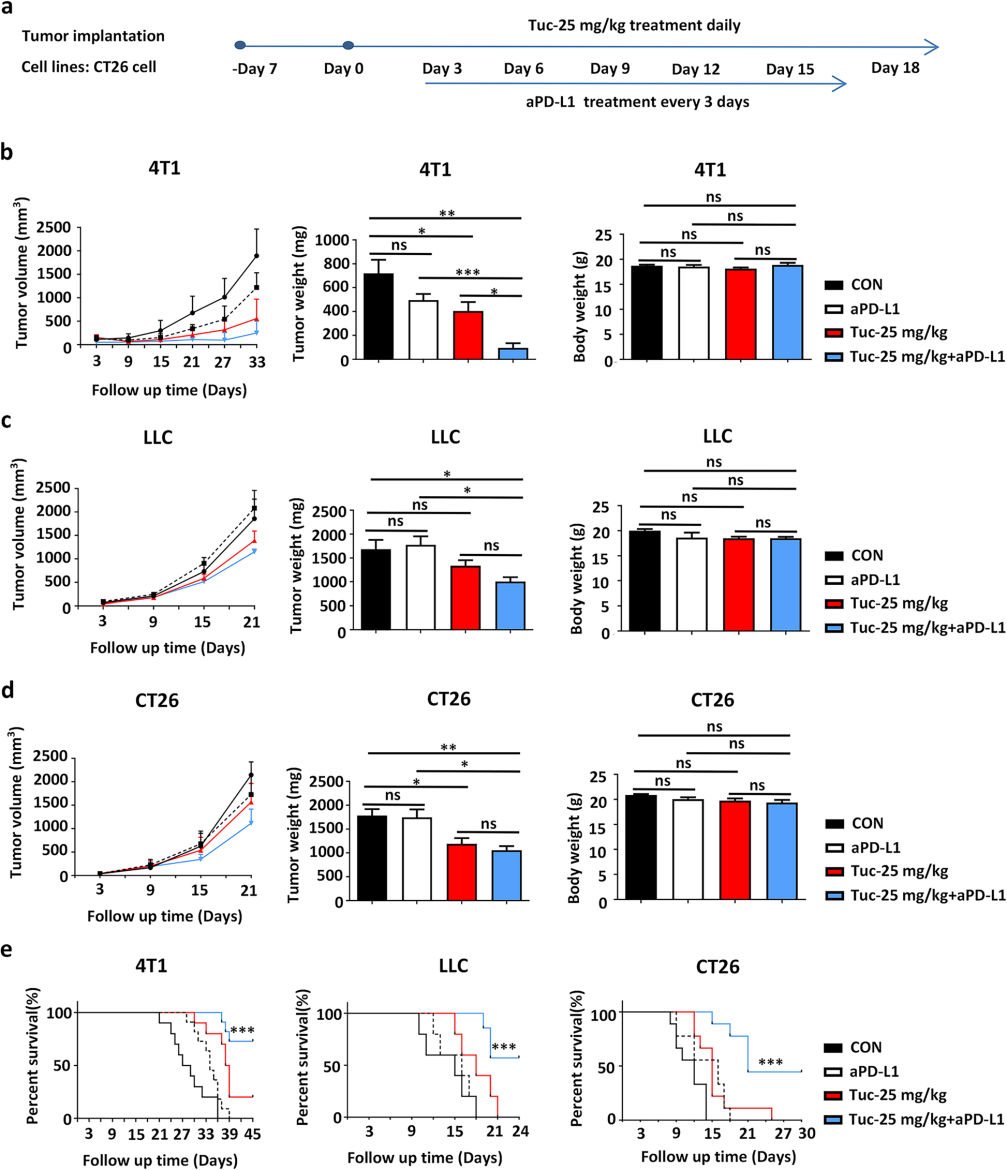
Tucidinostat improves the efficacy of checkpoint blockade and induces a durable response. **a** Schema of the experiment. Mouse 4T1, LLC, and CT26 cells (5 × 10^5^ cells) were engrafted into the flank of BALB/c or C57 BL/6 mice. When established tumors were palpable 7 days after tumor cell inoculation, mice were treated with vehicle (DMSO, *n*=7), tucidinostat (25 mg/kg, gavage, daily, *n*=7), aPD-L1 (200 μg, i.p. injection, once every 3 days, *n*=7), or combination (*n*=7). Tumor volume was measured with calipers every 3 days. **b** Tumor growth curves (left) of these mice at day 33 post-treatment initiation are shown in the 4T1 mouse tumor model. Tumor weight (middle) and mouse weight of these mice (right) at day 21 post-treatment initiation are shown. **c** Tumor growth curves (left), tumor weight (middle), and mouse weight (right) of these mice at day 21 post-treatment initiation are shown in the LLC mouse tumor model. **d** Tumor growth curves (left), tumor weight (middle), and mouse weight (right) of these mice at day 21 post-treatment initiation are shown in the CT26 mouse tumor model. **e** Kaplan-Meier survival curves of these mice are shown in 4T1, LLC, and CT26 mouse tumor models. The error bars indicate mean ± SEM. *P<0.05, **P<0.01, ***P<0.001 by one-way ANOVA or log-rank test. ns not significant, CON control group, Tuc tucidinostat, aPD-L1 anti-programmed cell death ligand 1 antibody

Because a robust antitumor immune response was induced by daily administration of 25 mg/kg tucidinostat, the treatment efficacy of tucidinostat combined with ICIs was further evaluated in three murine solid tumor models (4T1, LLC, and CT26). Accordingly, tumor-bearing mice were treated with tucidinostat (25 mg/kg) monotherapy through gavage, aPD-L1 (200 μg, every 3 days) monotherapy by intraperitoneal injection, tucidinostat plus aPD-L1, or vehicle control (Fig. 4a, Additional file 3: Fig. S3a). Tumor growth was significantly inhibited in the group treated with combination regimens compared to those treated with either single tucidinostat or vehicle control (Fig. 4b–d, Additional file 3: Fig. S3b-c). Notably, the combination of tucidinostat and aPD-L1 resulted in the significantly improved survival compared to vehicle control (Fig. 4e).

**Fig. 5:**
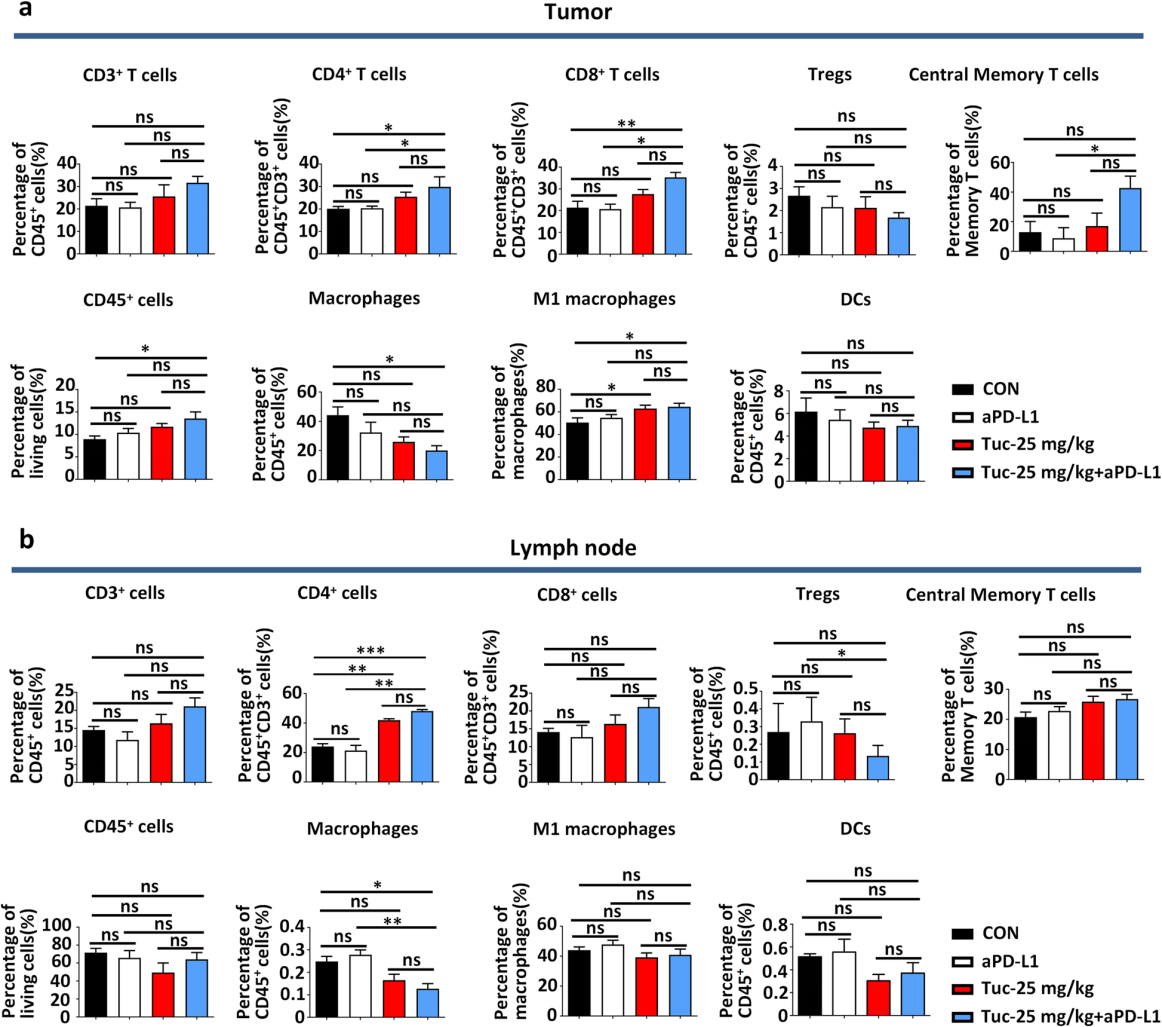
Tucidinostat and checkpoint blockade increase CD8^+^ T cell infiltration and M1 polarization in tumors. Flow cytometric quantification of lymphocytes (CD45^+^), total T cells (CD45^+^CD3^+^), CD4^+^ T cells (CD45^+^CD3^+^CD4^+^), CD8^+^ T cells (CD45^+^CD3^+^CD8^+^), Treg cells (CD45^+^CD3^+^CD4^+^CD25^+^), central memory T cells (CD3^+^CD4^+^CD44^+^CD62L^+^CD197^+^), macrophages (CD45^+^CD11b^+^F4/80^+^), M1 macrophages (CD45^+^CD11b^+^F4/80^+^/MHC-II^+^), and DCs (CD45^+^CD11b^-^CD11c^+^) in tumor parenchyma (**a**) and tumor drainage lymph nodes (**b**) from CT26 tumor-bearing mice on day 21 following different treatment groups (vehicle, tucidinostat, aPD-L1, and combination). The error bars indicate mean ± SEM. *P<0.05, **P<0.01, ***P<0.001 by one-way ANOVA. ns not significant, dLNs drainage lymph nodes, CON control group, Tuc tucidinostat, aPD-L1 anti-programmed cell death ligand 1 antibody

To investigate the effect of tucidinostat combined with aPD-L1 on TME, the changes observed in the immune cell populations upon treatment were analyzed. In subcutaneous CT26 tumors, the total amount of infiltrated CD45^+^ lymphocytes was increased after treatment with the combination therapy (Fig. 5a). Furthermore, it induced a significant increase in the proportion of tumor-infiltrating CD4^+^ and CD8^+^ T cells (Fig. 5a). In addition, immunohistochemical assays demonstrated an increase in the number of CD8^+^ T cells in tumor tissues collected from the combination therapy group (Additional file 4: Fig. S4a). Interestingly, CD8^+^ and CD4^+^ T cells from the combination therapy group exhibited a noticeable reduction in the expression of the exhaustion marker PD-1 (Additional file 4: Fig. S4b). Furthermore, the reductions in the proportion of tumor-infiltrating macrophages and increases in that of M1 macrophages were observed in the combination therapy group (Fig. 5a). The fluorescence-activated cell sorting (FACS) data of dLNs also confirmed that the antitumor activity and immune function were improved following the combination therapy. The percentages of CD4^+^ T cells significantly increased, whereas those of macrophages declined after treatment with tucidinostat plus aPD-L1 (Fig. 5b). Other immune cells such as myeloid-derived suppressor cells (MDSCs), dendritic cells (DCs), and natural killer (NK) cells were not significantly influenced in tumor tissues or dLNs (Fig. 5a, b, Additional file 4: Fig. S4c).

Furthermore, tucidinostat-induced changes in serum cytokine levels were also examined in CT26 tumor-bearing mice. In accordance with the changes observed in intratumoral expression of CD8^+^ T cells, serum CCL5 level was markedly increased after tucidinostat treatment alone as well as by the combination of tucidinostat plus aPD-L1 (Additional file 5: Fig. S5a-b). In addition, serum interferon-γ (IFN-γ) was enhanced in the combination therapy group compared with the vehicle control group. However, no changes were observed in serum interleukin-10 (IL-10) or tumor necrosis factor-α (TNF-α) (Additional file 5: Fig. S5c).

In general, these findings indicate that tucidinostat might significantly augment the antitumor immune response of aPD-L1 through CD8^+^ T cell infiltration and M1 macrophage polarization in solid tumor-bearing mice.

### CD8^+^ T cells are required for the induction of antitumor immunity by tucidinostat and aPD-L1 blockade

**Fig. 6:**
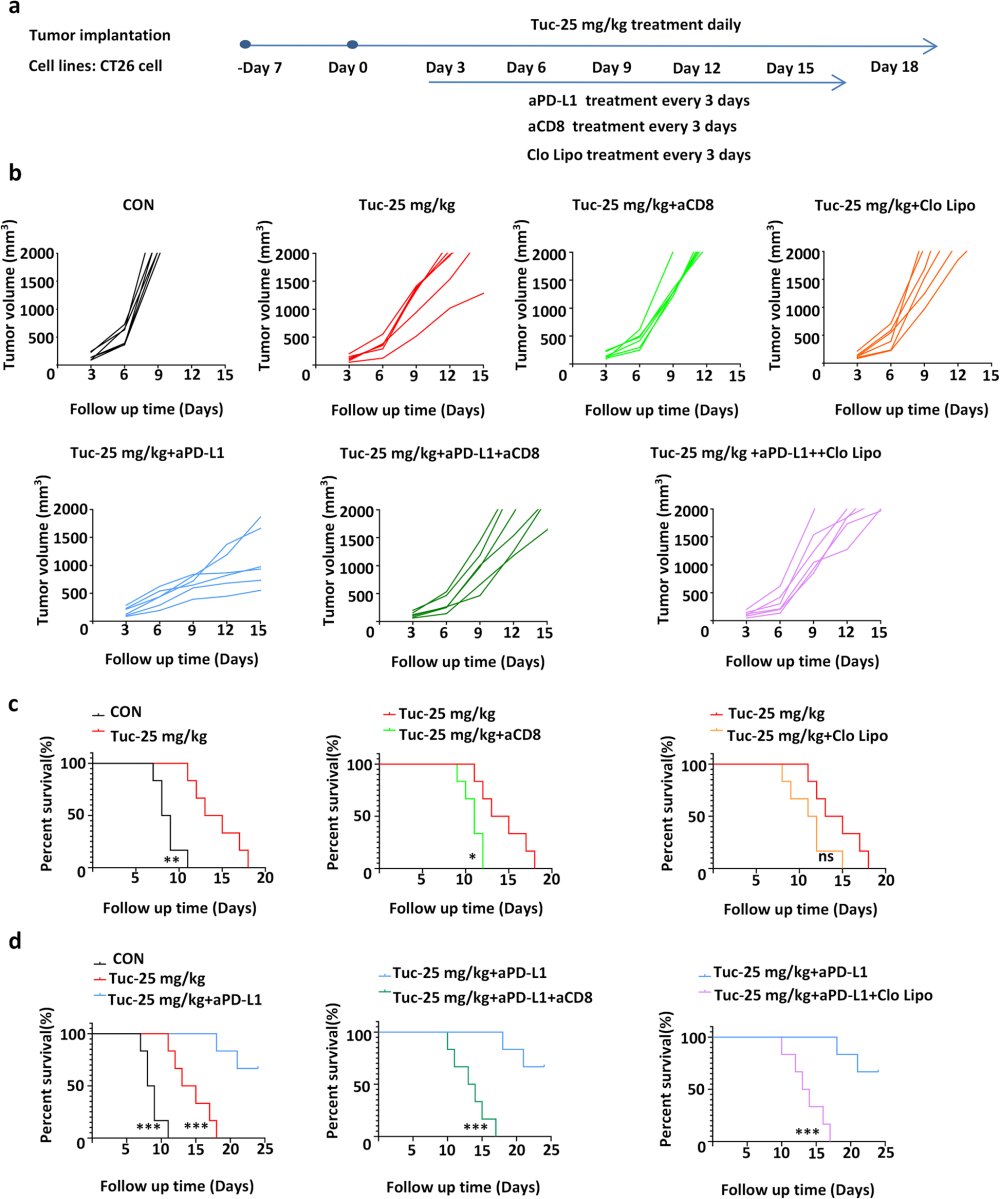
CD8^+^ T cells are required for antitumor immunity induced by tucidinostat and aPD-L1 blockade. **a** Schema of the experiment. Mouse CT26 cells (5 × 10^5^ cells) were engrafted into the flank of BALB/c mice. When established tumors were palpable 7 days after tumor cell inoculation, mice were treated with tucidinostat (25 mg/kg, gavage, daily, *n*=7) ± aPD-L1 (200 μg, i.p. injection, once every 3 days, *n*=7) regimen as indicated, in the presence or absence of aCD8 (CD8^+^ T depletion; 200 μg, i.p. injection, once every 3 days, *n*=7) or clodronate liposomes (macrophage depletion; 1.4 mg/20 g body weight, i.p. injection, once every 3 days, *n*=7). The following groups were included: vehicle, tucidinostat, tucidinostat+aCD8, tucidinostat+clodronate liposomes, tucidinostat+aPD-L1; tucidinostat+aPD-L1+aCD8, and tucidinostat+aPD-L1+clodronate liposomes. Tumor volume was measured with calipers every 3 days. **b** Tumor growth curves of these mice are shown in the CT26 mouse tumor model. **c** Survival differences between the tucidinostat group and the tucidinostat+aCD8/clodronate liposome group were evaluated. **d** Survival differences between the tucidinostat+aPD-L1 group and the tucidinostat+aPD-L1+aCD8/clodronate liposome group were evaluated. The error bars indicate mean ± SEM. *P<0.05, **P<0.01, ***P<0.001 by one-way ANOVA or log-rank test. ns not significant, CON control group, Tuc tucidinostat, aPD-L1 anti-programmed cell death ligand 1 antibody, aCD8 anti-CD8 antibody, Clo Lipo clodronate liposomes

As tucidinostat plus PD-L1 blockade resulted in tumor regression and flow cytometry profiling revealed enhanced CD8^+^ T cell infiltration, we hypothesized that the observed antitumor responses were mediated through CD8^+^ T cell populations. To further verify the contribution of CD8^+^ T cells to the enhanced antitumor efficacy of aPD-L1, we developed an in vivo CT26 mouse tumor model and utilized it for examining tumor responses following vehicle control, tucidinostat monotherapy ± CD8^+^ T cell depletion, and tucidinostat plus aPD-L1 combination therapy ± CD8^+^ T cell depletion. Upon pretreatment with an anti-CD8 antibody (aCD8), the degree of tumor shrinkage induced by both monotherapy and combination therapy was significantly reduced (Fig. 6a–d), supporting that tucidinostat potentiated the effects of PD-L1 blockade in vivo through CD8^+^ T cell-induced antitumor immune response.

In addition, macrophage depletion using clodronate liposomes also reversed the beneficial effects of combination treatment, suggesting that macrophages also contribute to tumor regression. However, macrophage depletion failed to reverse the antitumor response induced by tucidinostat alone (Fig. 6a–d). Therefore, macrophages might be necessary but not sufficient for the tumor suppression effect of tucidinostat.

### Tucidinostat increases the expression of the costimulatory molecules on human monocytes but fails to directly promote the transient activation of peripheral T cells

**Fig. 7:**
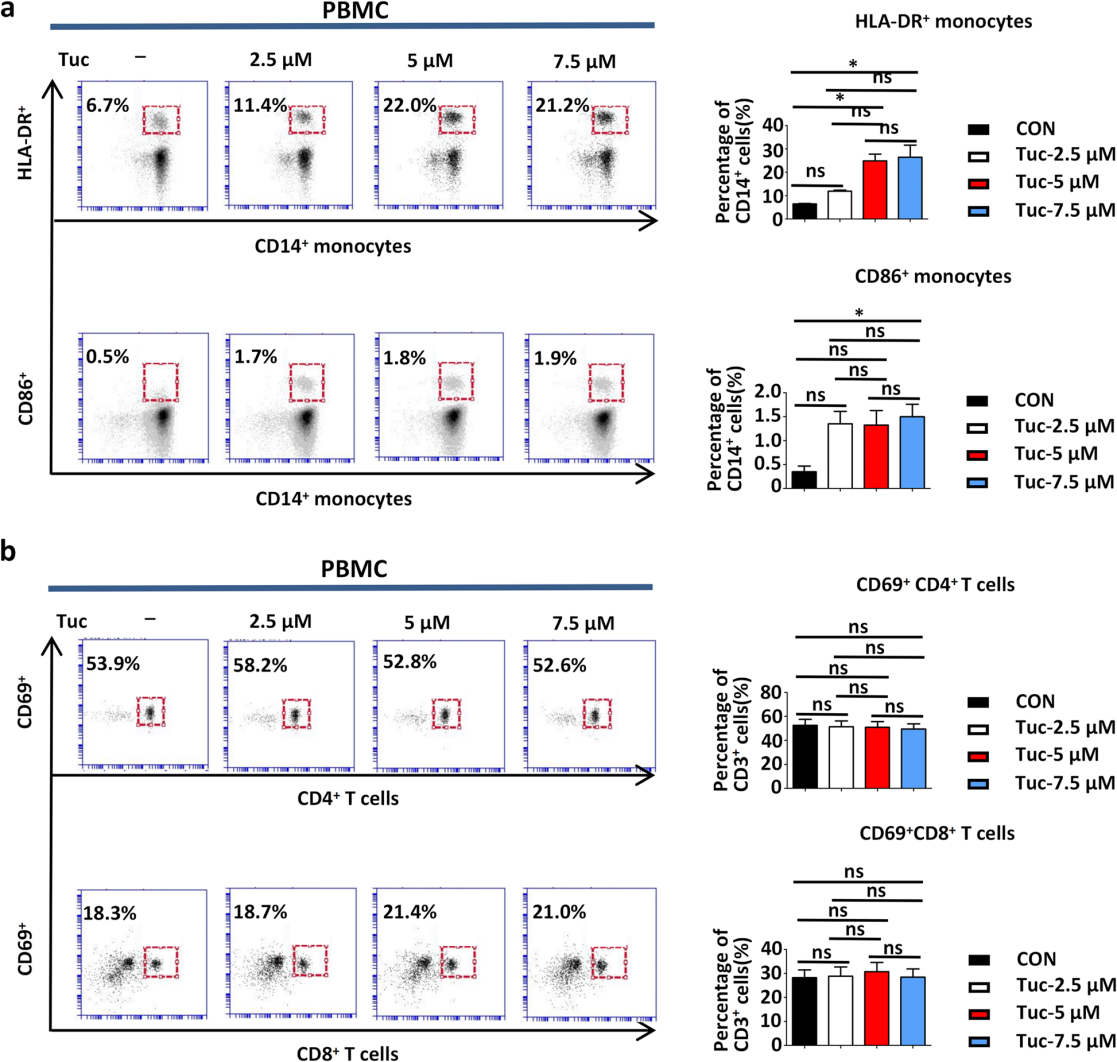
Tucidinostat increases the expression of the costimulatory molecules on human monocytes. **a** PBMCs from NSCLC patients (*n*=5) were cultured with increasing concentrations (2.5, 5, 7.5 μM) of tucidinostat for 24 h, respectively. Whereafter, the phenotype of peripheral blood monocytes (CD14^+^CD11b^+^) was assessed by FACS. Representative cytograms (left) or summary histograms (right) for the expression levels of HLA-DR and CD86 on gated monocytes within the PBMCs. **b** PBMCs from NSCLC patients were cultured with increasing concentrations (2.5, 5, 7.5 μM) of tucidinostat for 24 h, respectively. Whereafter, the phenotypes of CD4^+^ T cells (CD3^+^CD4^+^) and CD8^+^ T cells (CD3^+^CD8^+^) were assessed by FACS. Representative cytograms (left) or summary histograms (right) for the expression levels of CD69 on gated CD4^+^ and CD8^+^ T cells within the PBMCs. The error bars indicate mean ± SEM. *P<0.05, **P<0.01, ***P<0.001 by one-way ANOVA. ns not significant, CON control group, Tuc tucidinostat, PBMC peripheral blood mononuclear cell, NSCLC non-small cell lung cancer

Previous studies have reported that tucidinostat could alter antigen-presenting cell (APC) function by regulating inflammatory cytokine production in patients with immune thrombocytopenia [28]. We therefore explored whether tucidinostat could alter the antigen-presenting function of monocytes (CD14^+^CD11b^+^) among the peripheral blood mononuclear cells (PBMCs) of patients with non-small cell lung cancer (NSCLC). After 24-h treatment with tucidinostat, the surface expression of CD86 and HLA-DR on the monocyte fraction were significantly upregulated (Fig. 7a). These findings demonstrated the modulatory effects of tucidinostat on human monocytes and suggested that tucidinostat promotes these phenotypic changes, conferring enhanced antigen presentation and costimulatory capabilities. However, tucidinostat did not appear to activate T cells directly as no upregulation of the CD69 expression was observed on the conventional CD8^+^ or CD4^+^ T cells among PBMCs of patients with NSCLC (Fig. 7b).

## Discussion

HDACs consist of a large family of proteins categorized into five groups—class I (HDAC 1, 2, 3, 8), class IIa (HDAC 4, 5, 7, 9), class IIb (HDAC 6, 10), class III (Sirtuins), and class IV (HDAC 11) [18]. Aberrant HDAC expression occurs in most solid tumors and hematological cancers. The dysregulation of histone acetylation can lead to aberrant gene expression, which can activate oncogenes, inactivate tumor suppressors, inhibit programmed cell death, and mediate immune evasion, ultimately resulting in tumor progression [4, 16, 18, 21]. To date, four HDAC inhibitors, vorinostat, romidepsin, panobinostat, and belinostat, have been approved by the US Food and Drug Administration and are used for treating hematological cancers [45]. At present, most of the HDAC inhibitors in the clinic are pan-HDAC inhibitors. This broad-spectrum activity may also produce undesirable side effects. Therefore, the development of selective HDAC inhibitors may be useful for better understanding the critical events related to their therapeutic effects and for providing a rational basis to exploit synergistic interactions with other clinically effective agents.

Tucidinostat, a selective HDAC inhibitor having specificity for HDAC1, HDAC2, HDAC3, and HDAC10 subtypes, has been approved for the treatment of relapsed or refractory peripheral T cell lymphoma and is under clinical development globally for various other neoplastic and non-neoplastic diseases. Recently, tucidinostat has been approved by the National Medical Products Administration and is used for patients with advanced, hormone receptor-positive, HER2-negative breast cancer that progressed after previous endocrine therapy [30]. It might be the first HDAC inhibitor used for treating solid tumors in clinical settings. However, due to the grade 3 or 4 toxicities caused by it, tucidinostat might not effectively modulate TME. Therefore, the appropriate dose of tucidinostat should be defined for treating solid tumors. The mice were gavaged with tucidinostat daily at different dosages of tucidinostat after tumor cell inoculation. We demonstrated that tucidinostat at 25 mg/kg daily dosage could promote a rapid and sustained antitumor immune response through the preclinical mouse tumor model. Conversely, lower or opposite immunosuppressive effects were observed with the administration of lower or higher dosages. To date, few studies have reported regarding tucidinostat treatment optimization strategies for turning the TME of solid tumors from cold to hot. In this work, we mainly focused on exploring such optimization strategies and then delineating the possible underlying functional mechanisms both in vitro and in vivo.

Next, we demonstrated that the optimized dose tucidinostat could promote the secretion of several CD8^+^ T cell-attracting chemokines, especially that of CCL5. Although the role of CCL5/CCR5 axis in carcinogenesis is controversial [46], increasing evidence has demonstrated that constitutive CCL5 expression enables tumor immune recognition and enhances immunotherapy response via increased infiltration of CD8^+^ T cells into tumors [47–50]. Besides, high intratumoral expression of CCL5 is correlated with better prognosis and strongly correlated with intratumoral CD8A expression across multiple cancer types according to our analysis of TCGA datasets. It was reported that DNA methylation negatively regulates CCL5 expression in lung and colon cancers [51]. Our findings indicated an additional epigenetic mechanism wherein the selective histone acetylation inhibitor tucidinostat could also induce CCL5 expression in tumors through the NF-κB signaling pathway, leading to CD8^+^ T cell infiltration into tumors. Because tucidinostat elevated the secretion of CCL5 and other T cell-attracting chemokines in TME, we sought to demonstrate that the optimized dose of tucidinostat can promote a rapid and sustained antitumor immune response when used in combination with aPD-L1 using multiple preclinical mouse tumor models. The response was dependent on enhanced CD8^+^ T cell infiltration in TME and was abrogated upon CD8^+^ T cell depletion. Thus, tucidinostat with an optimized dose could alter TME and promote the migration and infiltration of CD8^+^ T cells into tumors, partially by increasing the activity of chemokine CCL5 *via* NF-κB signaling. CCL5 might indeed be secreted by some other inflammatory cells in TME following tucidinostat treatment, which is required to be testified in further studies.

Furthermore, we demonstrated that tucidinostat could also modulate M1 polarization of macrophages in solid tumors. It has been reported that the class IIa HDAC inhibitor TMP195 could alter the tumor microenvironment and reduce tumor burden and pulmonary metastases by modulating macrophage phenotype in a macrophage-dependent autochthonous mouse model of breast cancer. Furthermore, TMP195 induced the recruitment and differentiation of highly phagocytic and stimulatory macrophages within tumors [52]. In this study, it was seen that tucidinostat could directly promote M1 polarization of macrophages; moreover, the medium from tumor cells treated with tucidinostat could also induce M1 polarization of macrophages, suggesting that factors secreted by tumor cells in response to tucidinostat treatment could repolarize tumor-associated macrophages. The M1 macrophages, which have a role in mediating the destruction of tumor cells and facilitating the recruitment of Th1 cells, were also found to be highly sensitive to tucidinostat plus aPD-L1 treatment. Moreover, the antitumor effect was mostly abolished by macrophage depletion using clodronate liposomes. Therefore, tucidinostat could significantly promote M1 polarization of macrophages and increase the antitumor efficacy of aPD-L1 in vivo.

In addition, significant upregulation of MHC class II molecules, CD86 and HLA-DR, was observed with phenotypic changes associated with increased APC priming. This observation was consistent with the known positive correlation between MHC class II expression and PD-1/PD-L1 inhibitor response [53, 54]. In fact, several studies have identified MHC class II expression as a potential biomarker for PD-1/PD-L1 therapeutic response. Therefore, tucidinostat may sensitize tumors against aPD-1/aPD-L1 blockade, at least partly, by modulating the expression of MHC class II molecules. However, tucidinostat failed to directly promote the transient activation of peripheral T lymphocytes, which is in agreement with the findings we obtained using mouse models wherein tucidinostat altered T cell function by upregulating T cell-attracting chemokines, such as CCL5.

As we know, the success of pan-essential inhibitors suggests that targeting pan-essential genes will remain an important strategy for solid tumor therapeutics development. However, the broad requirement for HDAC activity in normal human tissues along with inhibitor polypharmacology made it likely that side effects should be limiting [55]. In this study, we chose the selective HDAC inhibitor tucidinostat which has been successfully used in clinical and found that the optimized dose of tucidinostat was seen to alter TME by promoting the infiltration of T cells *via* the activation of the NF-κB pathway and the subsequent release of immune-related cytokines such as CCL5. Moreover, the optimized dose of tucidinostat modulated M1 polarization of macrophages and dramatically potentiated the antitumor efficacy of PD-L1 blockade in solid tumors. Therefore, developing therapeutics that target pan-essential genes, such as HDACs, requires careful target prioritization and validation, dosing optimization, and combination strategies, which need in-depth research in the future.

## Conclusions

Collectively, our study demonstrated that the combined use of tucidinostat at an optimized dose and PD-L1 blockade may work synergistically to reduce tumor burden by enhancing the immune function. The finding provides a strong rationale for conducting clinical trials to investigate this combination therapy for overcoming ICI treatment resistance and achieving better clinical outcomes for patients with solid tumors.

## Supplementary Information

**Additional file 1. **The effect of tucidinostat on cell proliferation, apoptosis and hepatonephric function. **a** Comparison of cell proliferation of 4T1, LLC, and CT26 cells treated with different doses (2.5, 5, 7.5 μM) of tucidinostat for 24 h by CCK-8 assay. **b** Comparison of cell apoptosis of 4T1, LLC, and CT26 cells treated with different doses of tucidinostat (2.5, 5, 7.5 μM) for 6 h by Annexin V-FITC/PI assay. **c** Mouse CT26 cells (5 × 10^5^ cells) were engrafted into the flank of BALB/c mice. When established tumors were palpable 7 days after tumor cells inoculation, mice were treated with different doses (12.5, 25, 75 mg/kg, gavage, daily, *n*=5) of tucidinostat. Analysis on hepatonephric function on day 10 post treatment initiation. The serum ALT, AST, and BUN from peripheral blood were measured using ELISA kits. The error bars indicate mean ± SEM. *P<0.05, **P<0.01, ***P<0.001 by one-way ANOVA. ns: not significant. CON: control group; Tuc: tucidinostat; ALT: alanine transaminase; AST: aspartate aminotransferase; BUN: blood urea nitrogen.

**Additional file 2. **The function of tucidinostat on tumor immunity. **a** Functional annotation clustering of genes regulated in tumor from CT26 tumor-bearing mice on day 10 post treatment with tucidinostat (25 mg/kg, gavage, daily, *n*=3) or DMSO as vehicle control (DMSO, *n*=3). **b** Representative cytograms (left) or summary histograms (right) for the cell surface PD-L1 expression in CT26 cells following different doses (2.5, 5, 7.5 μM) of vorinostat, tucidinostat, and TMP-195 treatment for 24h. **c** Scatter plots showing the range of associations (r) with 95% CI and proportionality of expression levels for CD8A and CCL5 in three solid tumor types (BRCA, LUAD, and COAD) using TCGA database. **d** Kaplan-Meier survival curves for overall survival in three solid tumor types as stratified by CCL5 expression status using TCGA database. TCGA: the genome cancer atlas; BRCA: breast invasive carcinoma; LUAD: lung adenocarcinoma; COAD: colon adenocarcinoma; CON: control group; Vor: vorinostat; Tuc: tucidinostat; TMP: TMP-195.

**Additional file 3. **Tucidinostat plus checkpoint blockade induces improved therapeutic efficacy. **a** Schema of the experiment. For bioluminescence imaging (BLI) *in vivo*, mouse 4T1-luc (5 × 10^5^ cells) were engrafted into the flank of BALB/c mice. When established tumors were palpable 7 days after tumor cells inoculation, mice were treated with vehicle (DMSO, *n*=7), tucidinostat (25 mg/kg, gavage, daily, *n*=7), aPD-L1 (200 μg, i.p. injection, once every 3 days, *n*=7), or combination (*n*=7). Tumors volume were measured with calipers every three days. **b** Left: Comparison of tumor size from 4T1-Luc tumor-bearing mice on day 21 post treatment initiation. Middle: Luciferase imaging of living mice were measured using the Caliper IVIS Lumina III Live Imaging System. Right: Quantitative analysis of fluorescence intensity of the tumor from 4T1-Luc tumor-bearing mice on day 21 post treatment initiation. **c** HE staining of tumor from 4T1-Luc tumor-bearing mice on day 21 post treatment initiation. The error bars indicate mean ± SEM. *P<0.05, **P<0.01, ***P<0.001 by one-way ANOVA. ns: not significant. CON: control group; Vor: vorinostat; Tuc: tucidinostat; TMP: TMP-195; aPD-L1: anti-programmed cell death ligand 1 antibody; BLI: bioluminescence imaging.

**Additional file 4. **Tucidinostat plus checkpoint blockade induces significant antitumor immunity. **a** Schema of the experiment. Mouse CT26 cells (5 × 10^5^ cells) were engrafted into the flank of BALB/c mice. When established tumors were palpable 7 days after tumor cells inoculation, mice were treated with vehicle (DMSO, *n*=7), tucidinostat (25 mg/kg, gavage, daily, *n*=7), aPD-L1 (200 μg, i.p. injection, once every 3 days, *n*=7), or combination (*n*=7). The proportion of intratumoral CD8^+^ T cells by IHC from CT26 tumor-bearing mice on day 21 post treatment initiation. **b** Flow cytometric quantification of PD-1 in CD4^+^ T cells (CD45^+^CD3^+^CD4^+^) and CD8^+^ T cells (CD45^+^CD3^+^CD8^+^) in tumor parenchyma and tumor drainage lymph nodes from CT26 tumor-bearing mice on day 21 post treatment initiation. **c** Flow cytometric quantification of MDSCs (CD45^+^CD11b^+^Gr-1^+^), active DC cells (CD45^+^CD11b^-^CD11c^+^CD86^+^), Effective Memory T cells (CD3^+^CD4^+^CD44^+^CD62L^-^), and NK cells (CD45^+^CD3^-^CD49b^+^) in tumor parenchyma and tumor drainage lymph nodes from CT26 tumor-bearing mice on day 21 post treatment initiation. The error bars indicate mean ± SEM. *P<0.05, **P<0.01, ***P<0.001 by one-way ANOVA. ns: not significant. CON: control group; Tuc: tucidinostat; aPD-L1: anti-programmed cell death ligand 1 antibody.

**Additional file 5. **Tucidinostat plus checkpoint blockade induces CCL5 secretion. **a** Mouse CT26 cells (5 × 10^5^ cells) were engrafted into the flank of BALB/c mice. When established tumors were palpable 7 days after tumor cells inoculation, mice were treated with different doses (12.5, 25, 75 mg/kg, gavage, daily, *n*=5) of tucidinostat. The expression of CCL5 in peripheral blood serum from CT26 tumor-bearing mice on day 10 post treatment initiation. Mouse CT26 cells (5 × 10^5^ cells) were engrafted into the flank of BALB/c mice. When established tumors were palpable 7 days after tumor cells inoculation, mice were treated with vehicle (DMSO, *n*=7), tucidinostat (25 mg/kg, gavage, daily, *n*=7), aPD-L1 (200 μg, i.p. injection, once every 3 days, *n*=7), or combination (*n*=7). The expression of CCL5 **b**, IFN-γ, TNF-α, and IL-10 **c** in peripheral blood serum from CT26 tumor-bearing mice on day 21 post treatment initiation. The error bars indicate mean ± SEM. *P<0.05, **P<0.01, ***P<0.001 by one-way ANOVA. ns: not significant. CON: control group; Tuc: tucidinostat; aPD-L1: anti-programmed cell death ligand 1 antibody.

**Additional file 6.** The original blots.

## Data Availability

All data relevant to the study are included in the article or uploaded as supplemental information.

## Abbreviations

aCD8: Anti-CD8 antibody
ALT: Alanine transaminase
APC: Antigen-presenting cell
aPD-L1: Anti-programmed cell death ligand 1 antibody
Arg 1: Arginase-1
AST: Aspartate aminotransferase
BLI: Bioluminescence imaging
BMDM: Bone marrow-derived macrophage
BRCA: Breast invasive carcinoma
BUN: Blood urea nitrogen
CCL5: C-C motif chemokine ligand 5
Clo Lipo: Clodronate liposomes
COAD: Colon adenocarcinoma
CON: Control group
CXCL10: C-X-C motif chemokine ligand 10
CXCL9: C-X-C motif chemokine ligand 9
DCs: Dendritic cells
dLNs: Drainage lymph nodes
FACS: Fluorescence-activated cell sorting
HDAC: Histone deacetylase
ICIs: Immune checkpoint inhibitors
IFN-γ: Interferon-γ
IL-10: Interleukin-10
iNOS: Inducible nitric oxide synthase
LLC: Lewis lung cancer
LUAD: Lung adenocarcinoma
mAb: Monoclonal antibody
MDSC: Myeloid-derived suppressor cell
NK cell: Natural killer cell
ns: Not significant
NSCLC: Non-small cell lung cancer
PBMC: Peripheral blood mononuclear cell
PD-1: Programmed cell death protein 1
pNF-κB p65: phospho-p65-NF-κB
PLT: Platelet
RBC: Red blood cell
TCGA: The Genome Cancer Atlas
TCM: Tumor-conditioned medium
TME: Tumor microenvironment
TMP: TMP-195
TNF-α: Tumor necrosis factor-α
Tuc: Tucidinostat
Vor: Vorinostat
WBC: White blood cell
