# Supplementary figures and images for: Optimized dose selective HDAC inhibitor tucidinostat overcomes anti-PD-L1 antibody resistance in experimental solid tumors
Source: BMC Med. 2022 Nov 9;20:435. doi: 10.1186/s12916-022-02598-5 (PMC9648046; doi:10.1186/s12916-022-02598-5)

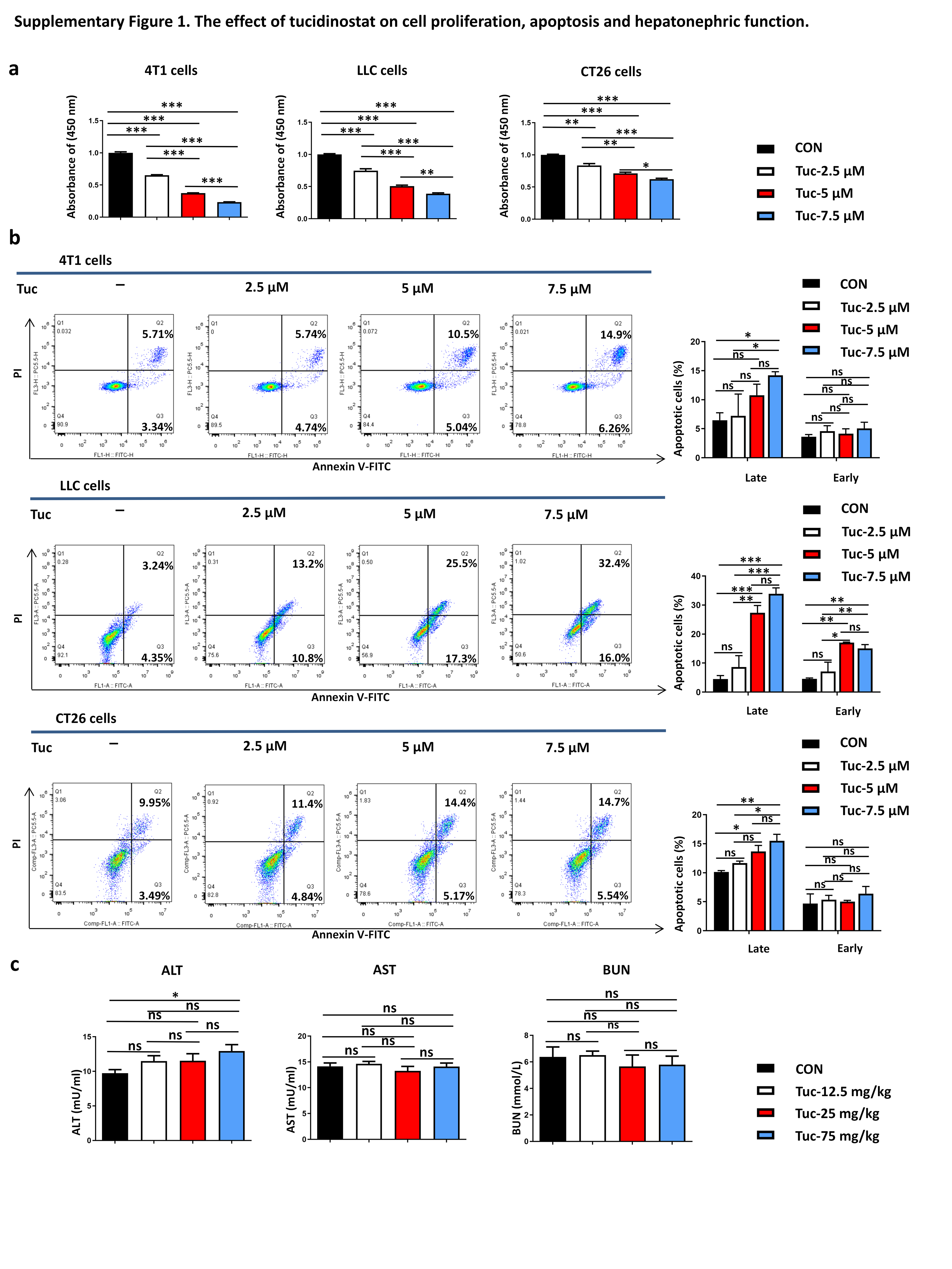

Supplement: Supplementary file 1 — Additional file 1. The effect of tucidinostat on cell proliferation, apoptosis and hepatonephric function. a Comparison of cell proliferation of 4T1, LLC, and CT26 cells treated with different doses (2.5, 5, 7.5 μM) of tucidinostat for 24 h by CCK-8 assay. b Comparison of cell apoptosis of 4T1, LLC, and CT26 cells treated with different doses of tucidinostat (2.5, 5, 7.5 μM) for 6 h by Annexin V-FITC/PI assay. c Mouse CT26 cells (5 × 10^5^ cells) were engrafted into the flank of BALB/c mice. When established tumors were palpable 7 days after tumor cells inoculation, mice were treated with different doses (12.5, 25, 75 mg/kg, gavage, daily, n=5) of tucidinostat. Analysis on hepatonephric function on day 10 post treatment initiation. The serum ALT, AST, and BUN from peripheral blood were measured using ELISA kits. The error bars indicate mean ± SEM. *P<0.05, **P<0.01, ***P<0.001 by one-way ANOVA. ns: not significant. CON: control group; Tuc: tucidinostat; ALT: alanine transaminase; AST: aspartate aminotransferase; BUN: blood urea nitrogen. [file 12916_2022_2598_MOESM1_ESM.tif]

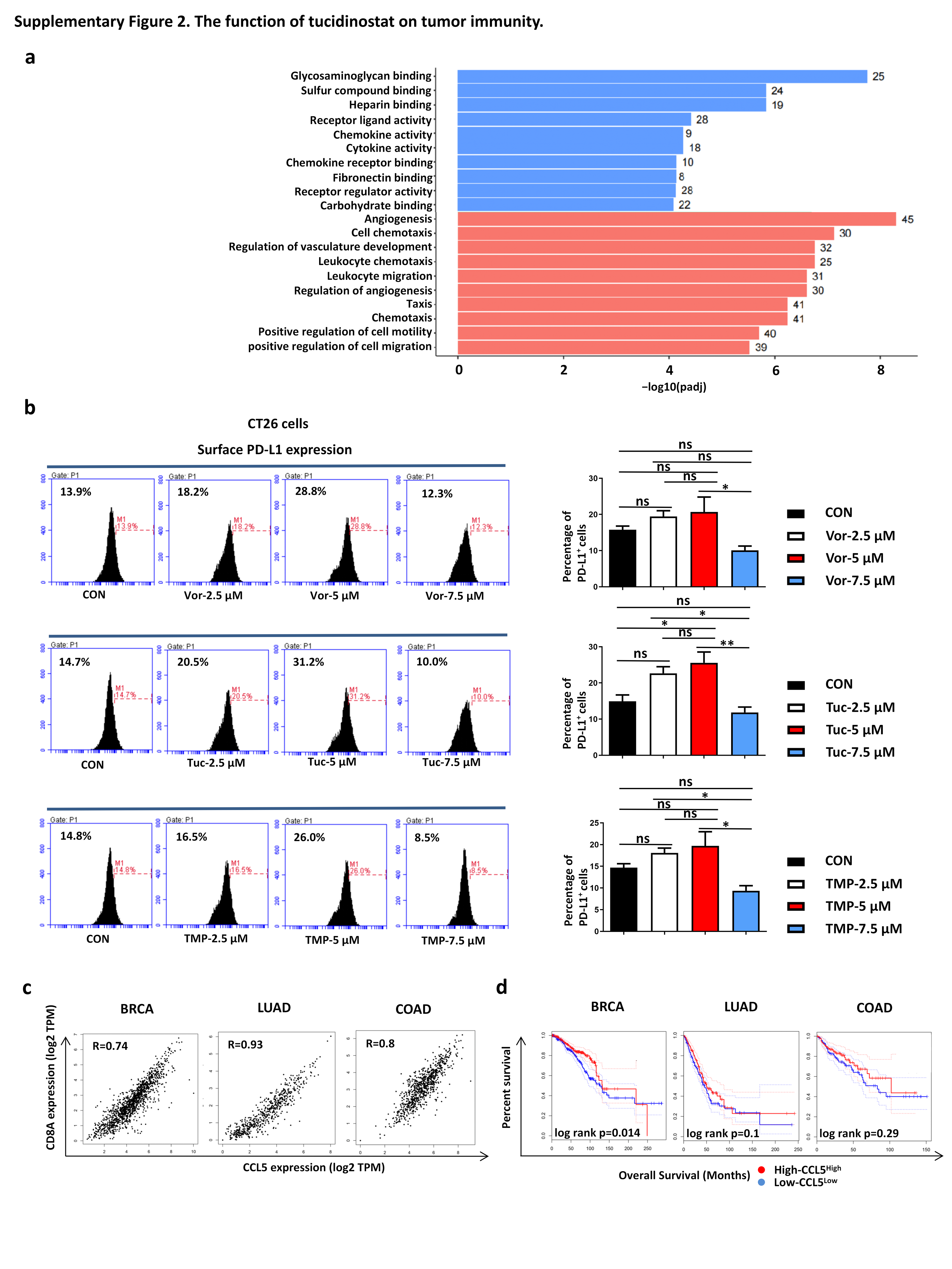

Supplement: Supplementary file 2 — Additional file 2. The function of tucidinostat on tumor immunity. a Functional annotation clustering of genes regulated in tumor from CT26 tumor-bearing mice on day 10 post treatment with tucidinostat (25 mg/kg, gavage, daily, n=3) or DMSO as vehicle control (DMSO, n=3). b Representative cytograms (left) or summary histograms (right) for the cell surface PD-L1 expression in CT26 cells following different doses (2.5, 5, 7.5 μM) of vorinostat, tucidinostat, and TMP-195 treatment for 24h. c Scatter plots showing the range of associations (r) with 95% CI and proportionality of expression levels for CD8A and CCL5 in three solid tumor types (BRCA, LUAD, and COAD) using TCGA database. d Kaplan-Meier survival curves for overall survival in three solid tumor types as stratified by CCL5 expression status using TCGA database. TCGA: the genome cancer atlas; BRCA: breast invasive carcinoma; LUAD: lung adenocarcinoma; COAD: colon adenocarcinoma; CON: control group; Vor: vorinostat; Tuc: tucidinostat; TMP: TMP-195. [file 12916_2022_2598_MOESM2_ESM.tif]

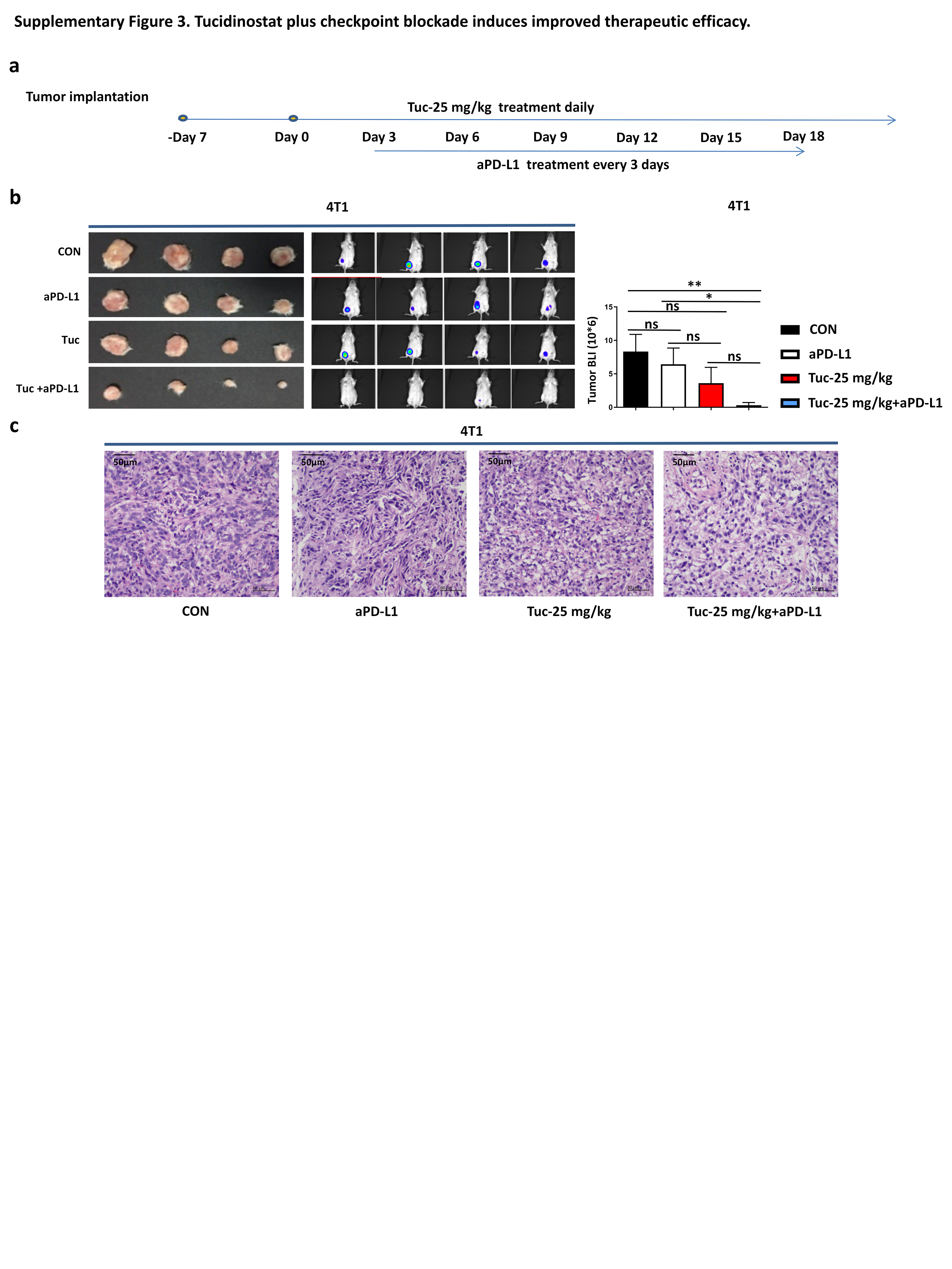

Supplement: Supplementary file 3 — Additional file 3. Tucidinostat plus checkpoint blockade induces improved therapeutic efficacy. a Schema of the experiment. For bioluminescence imaging (BLI) in vivo, mouse 4T1-luc (5 × 10^5^ cells) were engrafted into the flank of BALB/c mice. When established tumors were palpable 7 days after tumor cells inoculation, mice were treated with vehicle (DMSO, n=7), tucidinostat (25 mg/kg, gavage, daily, n=7), aPD-L1 (200 μg, i.p. injection, once every 3 days, n=7), or combination (n=7). Tumors volume were measured with calipers every three days. b Left: Comparison of tumor size from 4T1-Luc tumor-bearing mice on day 21 post treatment initiation. Middle: Luciferase imaging of living mice were measured using the Caliper IVIS Lumina III Live Imaging System. Right: Quantitative analysis of fluorescence intensity of the tumor from 4T1-Luc tumor-bearing mice on day 21 post treatment initiation. c HE staining of tumor from 4T1-Luc tumor-bearing mice on day 21 post treatment initiation. The error bars indicate mean ± SEM. *P<0.05, **P<0.01, ***P<0.001 by one-way ANOVA. ns: not significant. CON: control group; Vor: vorinostat; Tuc: tucidinostat; TMP: TMP-195; aPD-L1: anti-programmed cell death ligand 1 antibody; BLI: bioluminescence imaging. [file 12916_2022_2598_MOESM3_ESM.tif]

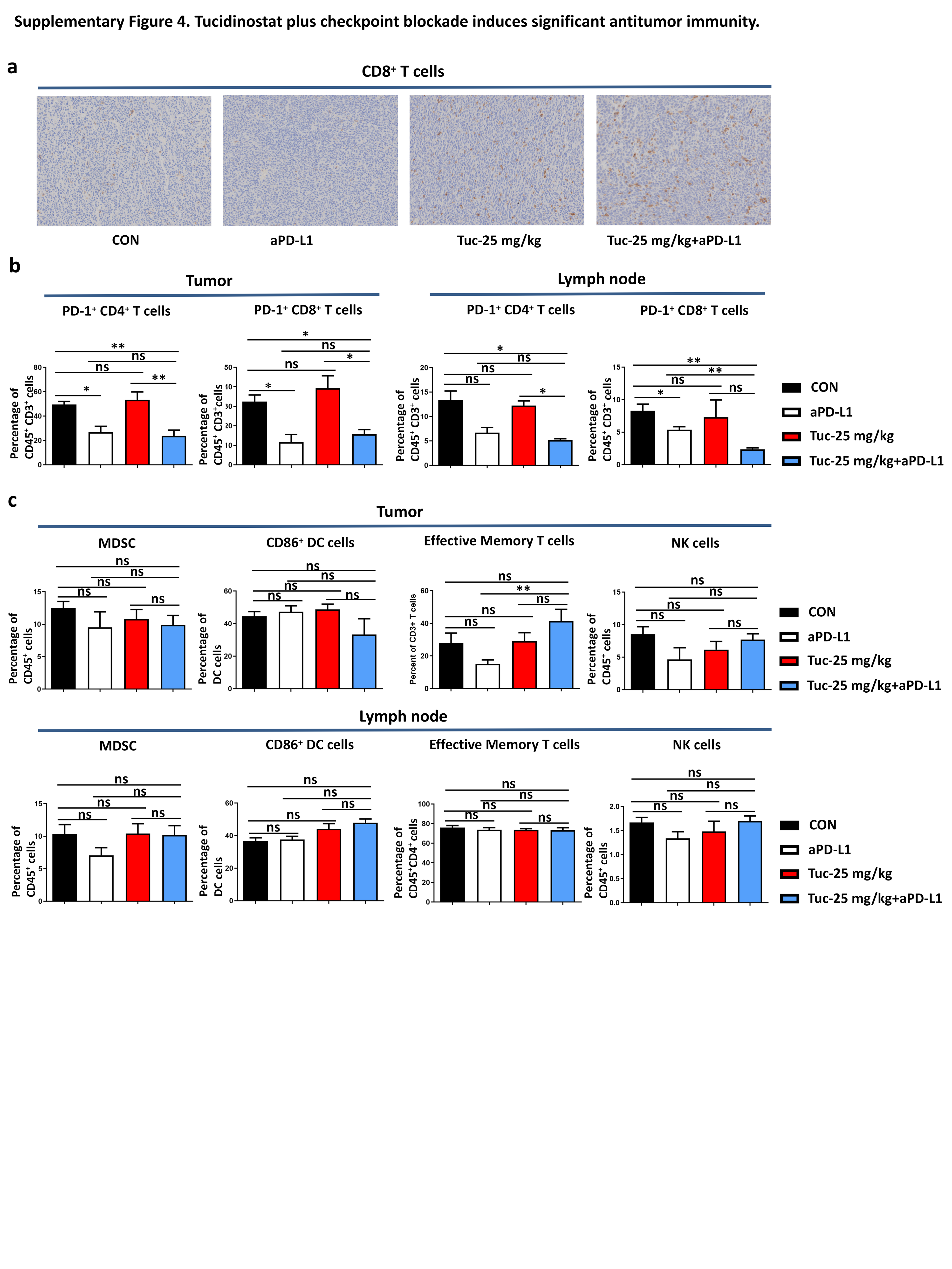

Supplement: Supplementary file 4 — Additional file 4. Tucidinostat plus checkpoint blockade induces significant antitumor immunity. a Schema of the experiment. Mouse CT26 cells (5 × 10^5^ cells) were engrafted into the flank of BALB/c mice. When established tumors were palpable 7 days after tumor cells inoculation, mice were treated with vehicle (DMSO, n=7), tucidinostat (25 mg/kg, gavage, daily, n=7), aPD-L1 (200 μg, i.p. injection, once every 3 days, n=7), or combination (n=7). The proportion of intratumoral CD8+ T cells by IHC from CT26 tumor-bearing mice on day 21 post treatment initiation. b Flow cytometric quantification of PD-1 in CD4+ T cells (CD45+CD3+CD4+) and CD8+ T cells (CD45+CD3+CD8+) in tumor parenchyma and tumor drainage lymph nodes from CT26 tumor-bearing mice on day 21 post treatment initiation. c Flow cytometric quantification of MDSCs (CD45+CD11b+Gr-1+), active DC cells (CD45+CD11b-CD11c+CD86+), Effective Memory T cells (CD3+CD4+CD44+CD62L-), and NK cells (CD45+CD3-CD49b+) in tumor parenchyma and tumor drainage lymph nodes from CT26 tumor-bearing mice on day 21 post treatment initiation. The error bars indicate mean ± SEM. *P<0.05, **P<0.01, ***P<0.001 by one-way ANOVA. ns: not significant. CON: control group; Tuc: tucidinostat; aPD-L1: anti-programmed cell death ligand 1 antibody. [file 12916_2022_2598_MOESM4_ESM.tif]

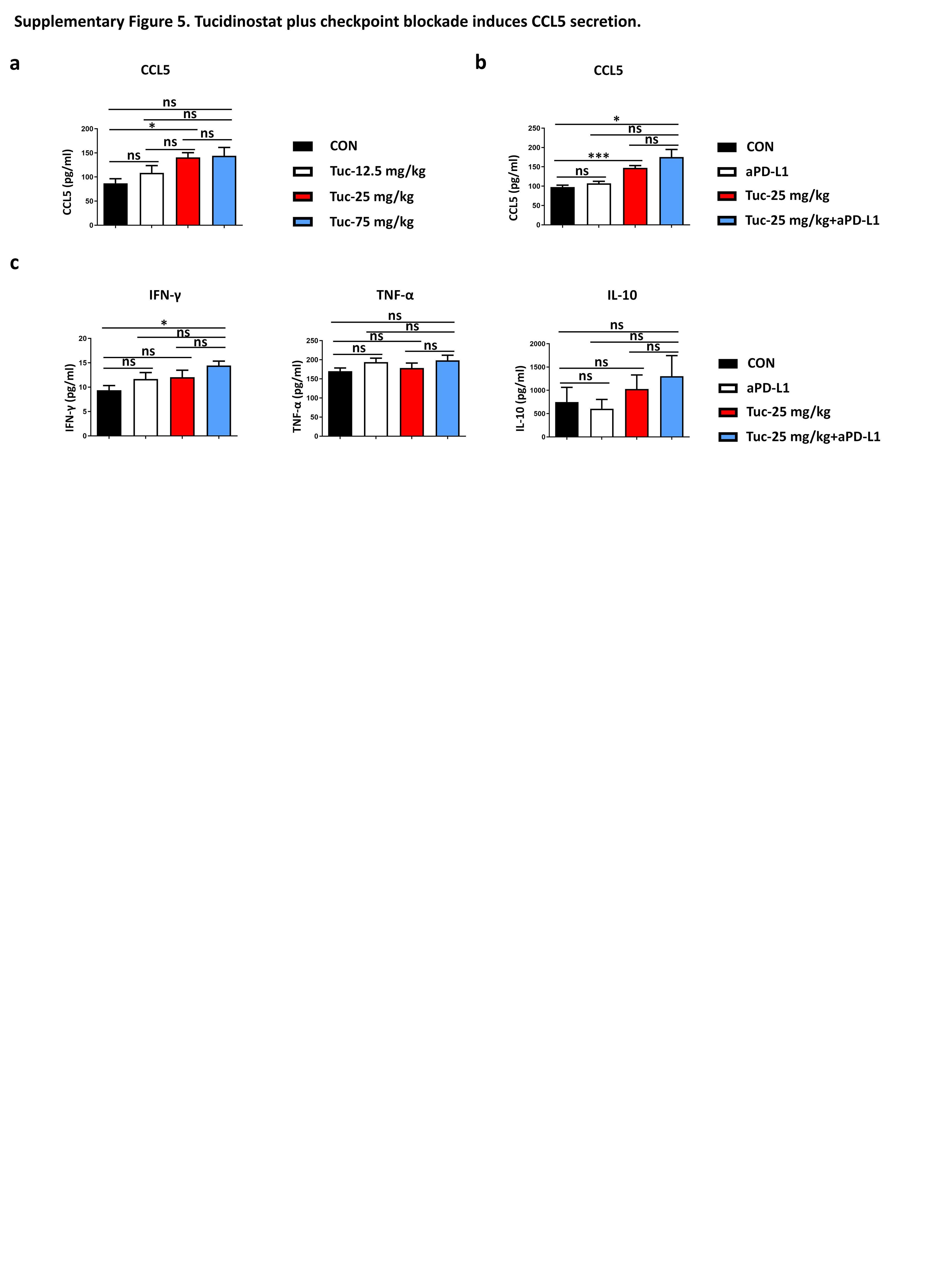

Supplement: Supplementary file 5 — Additional file 5. Tucidinostat plus checkpoint blockade induces CCL5 secretion. a Mouse CT26 cells (5 × 10^5^ cells) were engrafted into the flank of BALB/c mice. When established tumors were palpable 7 days after tumor cells inoculation, mice were treated with different doses (12.5, 25, 75 mg/kg, gavage, daily, n=5) of tucidinostat. The expression of CCL5 in peripheral blood serum from CT26 tumor-bearing mice on day 10 post treatment initiation. Mouse CT26 cells (5 × 10^5^ cells) were engrafted into the flank of BALB/c mice. When established tumors were palpable 7 days after tumor cells inoculation, mice were treated with vehicle (DMSO, n=7), tucidinostat (25 mg/kg, gavage, daily, n=7), aPD-L1 (200 μg, i.p. injection, once every 3 days, n=7), or combination (n=7). The expression of CCL5 b, IFN-γ, TNF-α, and IL-10 c in peripheral blood serum from CT26 tumor-bearing mice on day 21 post treatment initiation. The error bars indicate mean ± SEM. *P<0.05, **P<0.01, ***P<0.001 by one-way ANOVA. ns: not significant. CON: control group; Tuc: tucidinostat; aPD-L1: anti-programmed cell death ligand 1 antibody. [file 12916_2022_2598_MOESM5_ESM.tif]
