# Supplementary material for: Optimized dose selective HDAC inhibitor tucidinostat overcomes anti-PD-L1 antibody resistance in experimental solid tumors
Source: BMC Med. 2022 Nov 9;20:435. doi: 10.1186/s12916-022-02598-5 (PMC9648046; doi:10.1186/s12916-022-02598-5)
Supplement: Supplementary file 6 — Additional file 6. The original blots. [file 12916_2022_2598_MOESM6_ESM.pptx]

## Slide 1
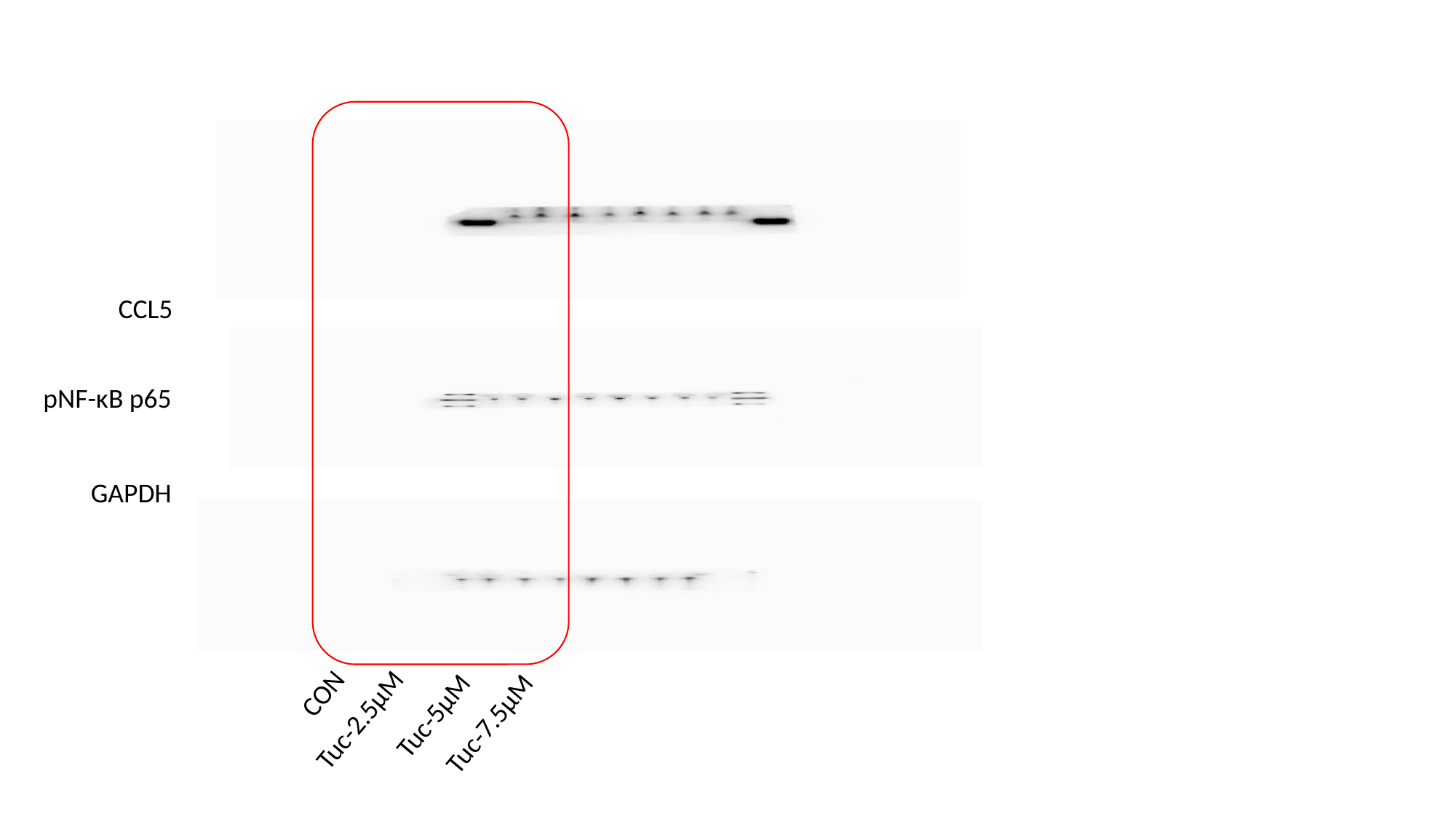

CCL5
pNF-κB p65
GAPDH
CON
Tuc-2.5μM
Tuc-5μM
Tuc-7.5μM

## Slide 2
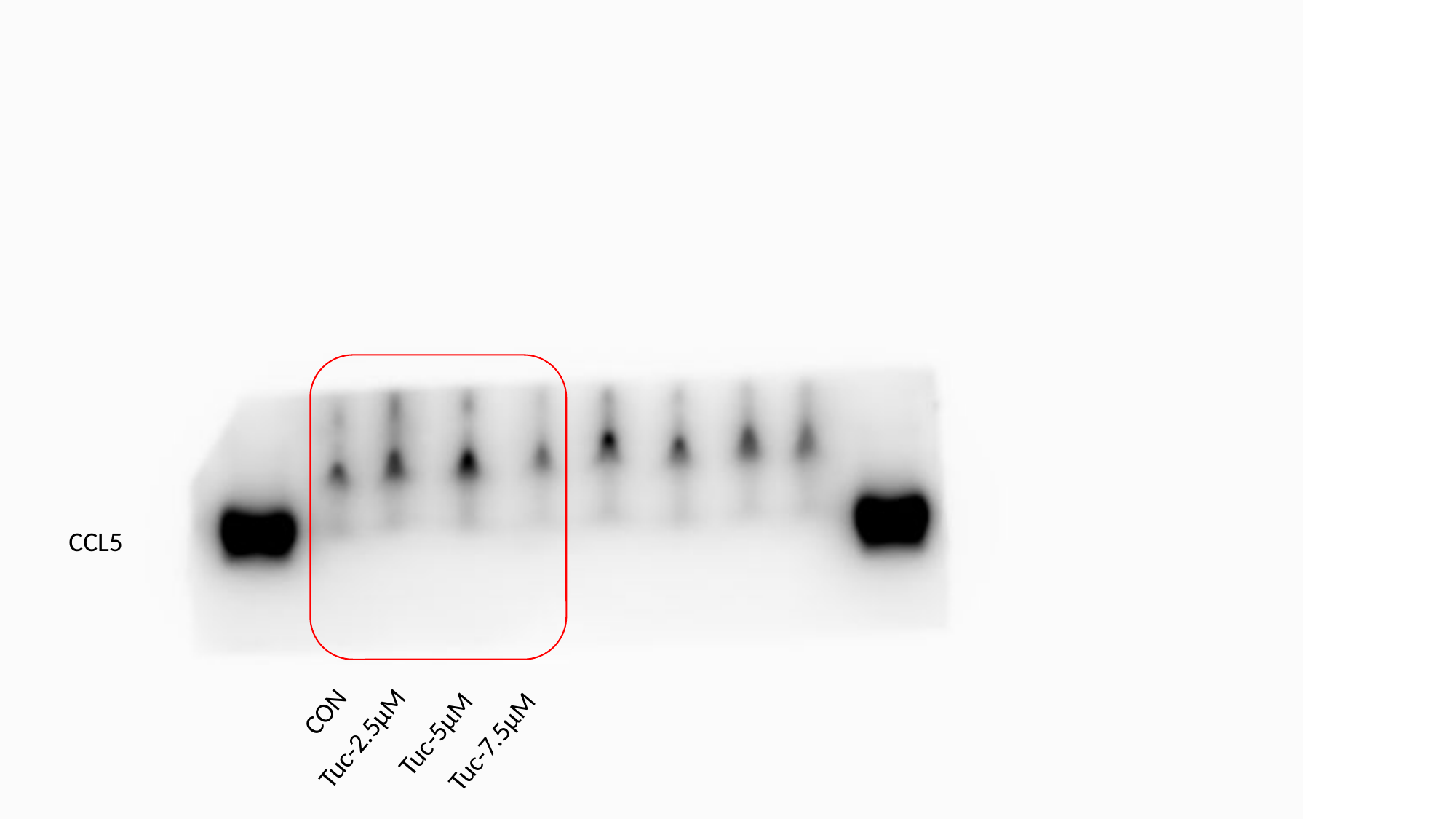

CCL5
CON
Tuc-2.5μM
Tuc-5μM
Tuc-7.5μM

## Slide 3
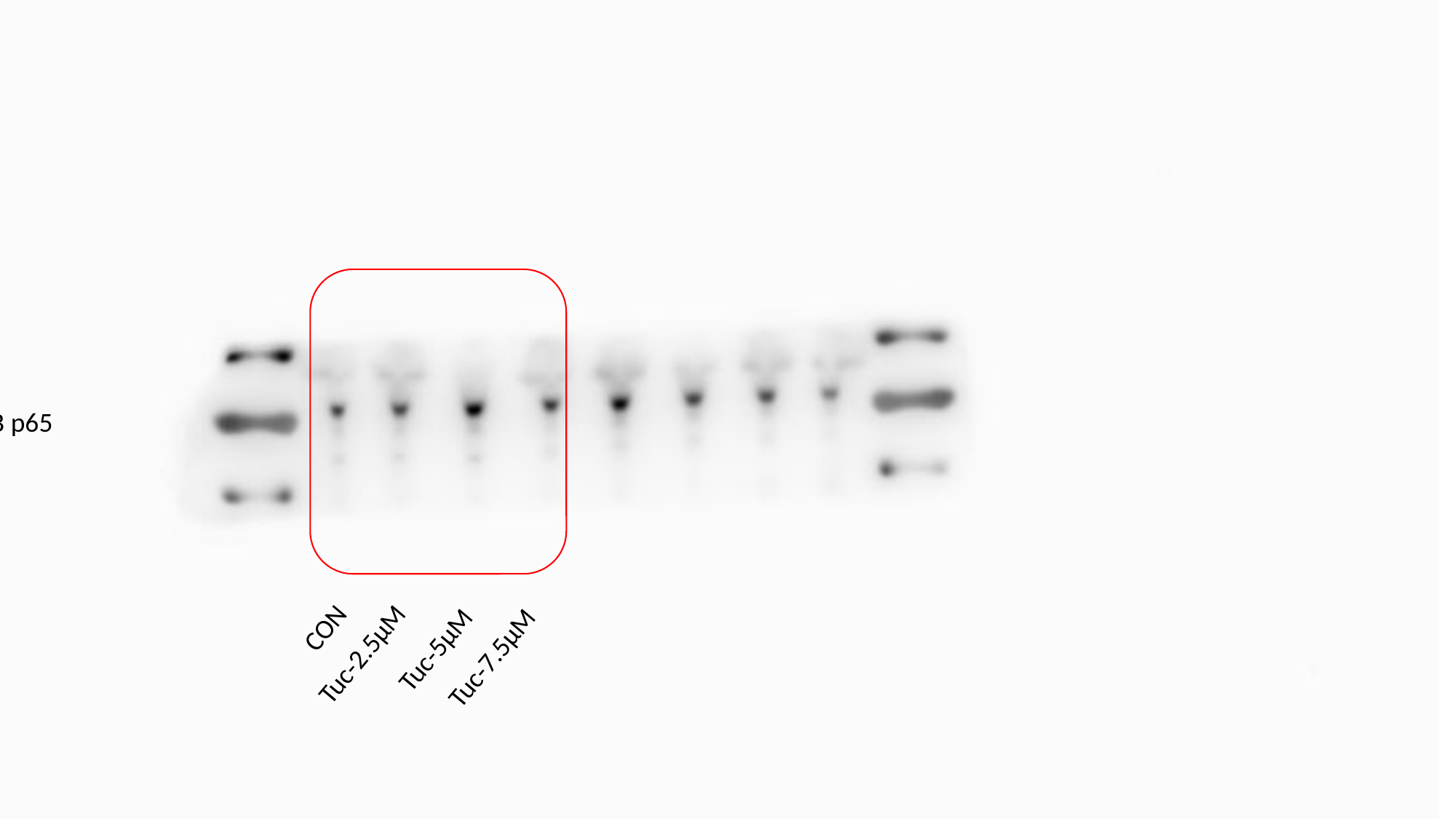

pNF-κB p65
CON
Tuc-2.5μM
Tuc-5μM
Tuc-7.5μM

## Slide 4
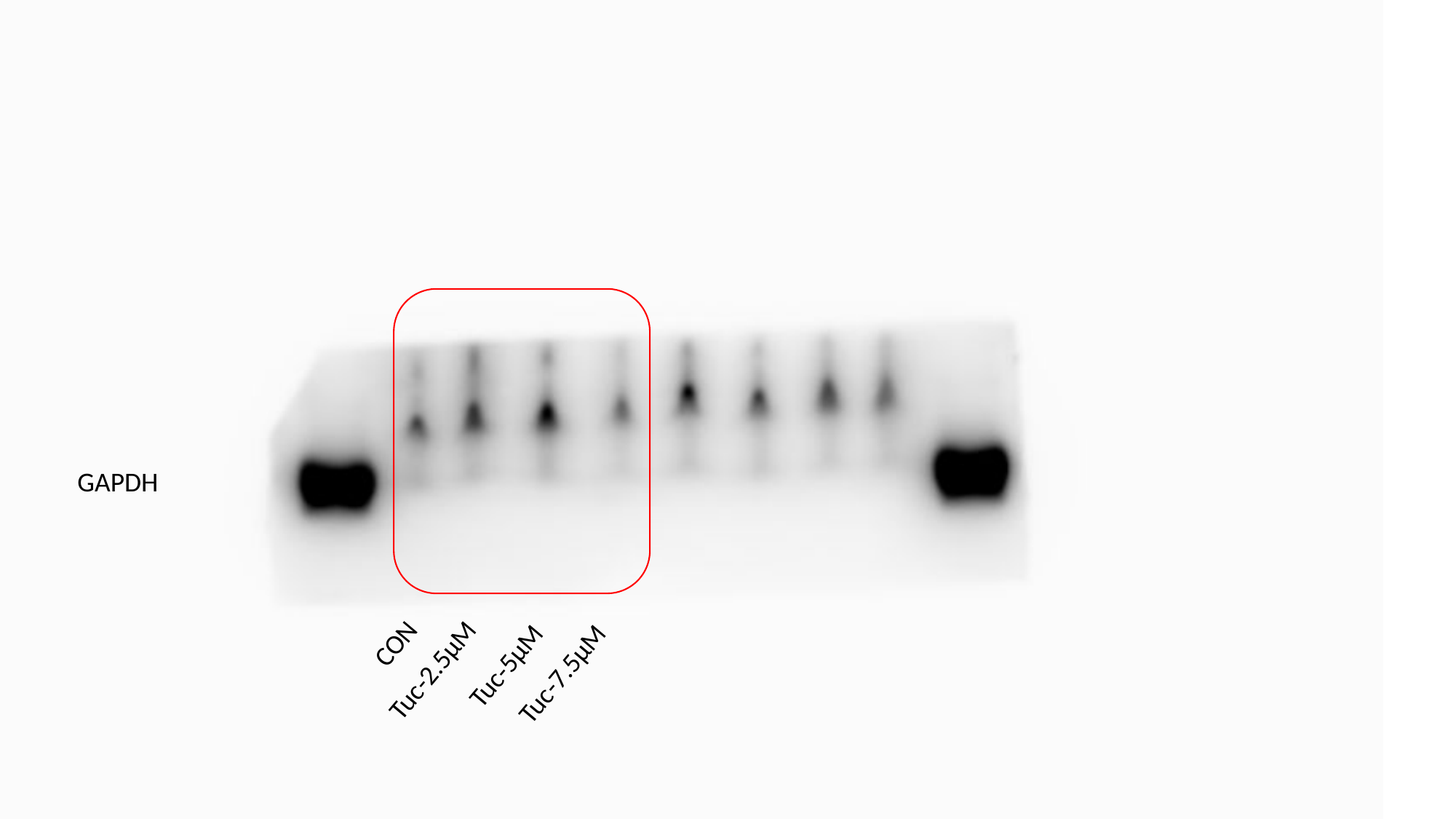

GAPDH
CON
Tuc-2.5μM
Tuc-5μM
Tuc-7.5μM

## Slide 5
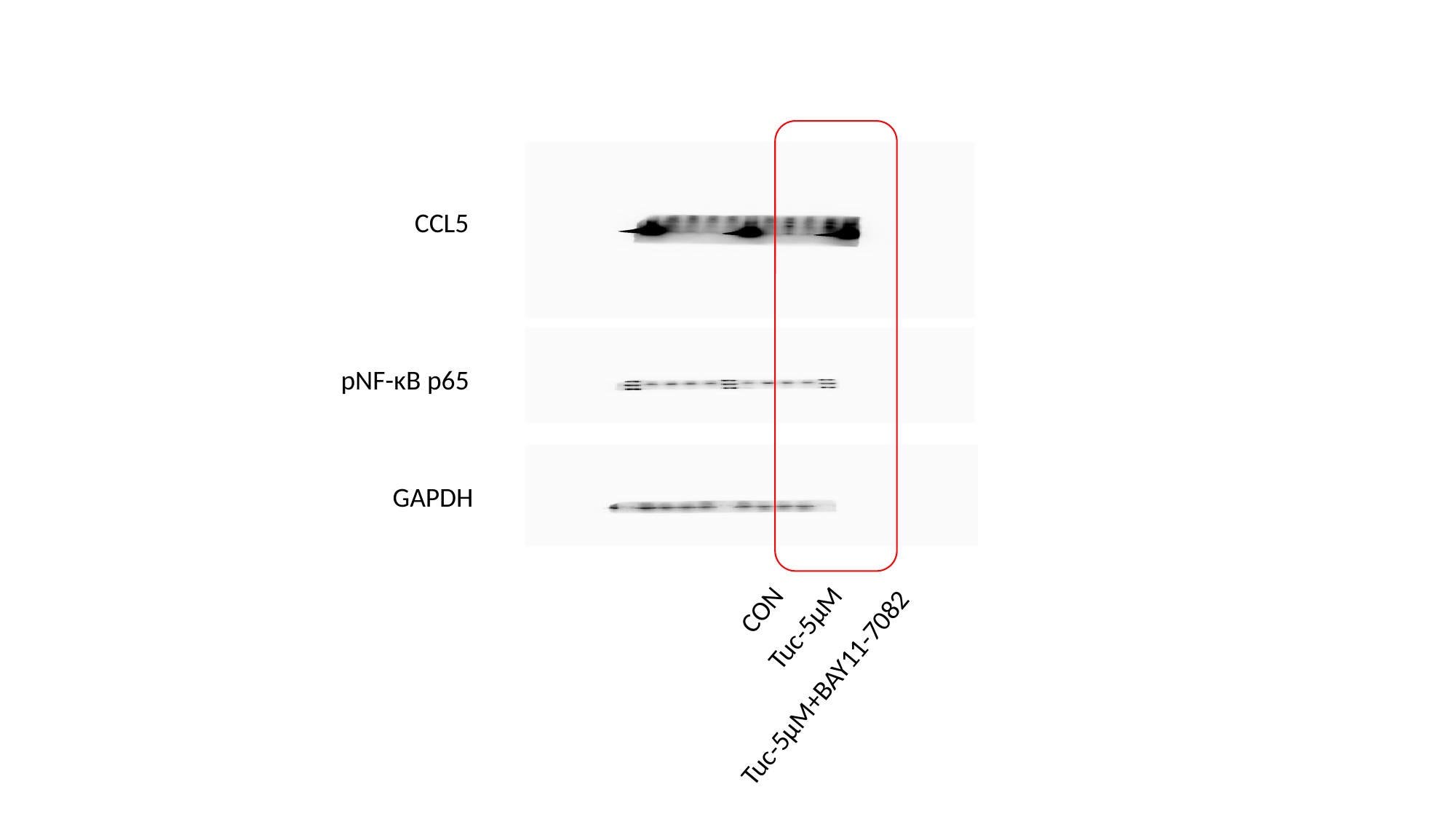

CCL5
pNF-κB p65
GAPDH
CON
Tuc-5μM
Tuc-5μM+BAY11-7082

## Slide 6
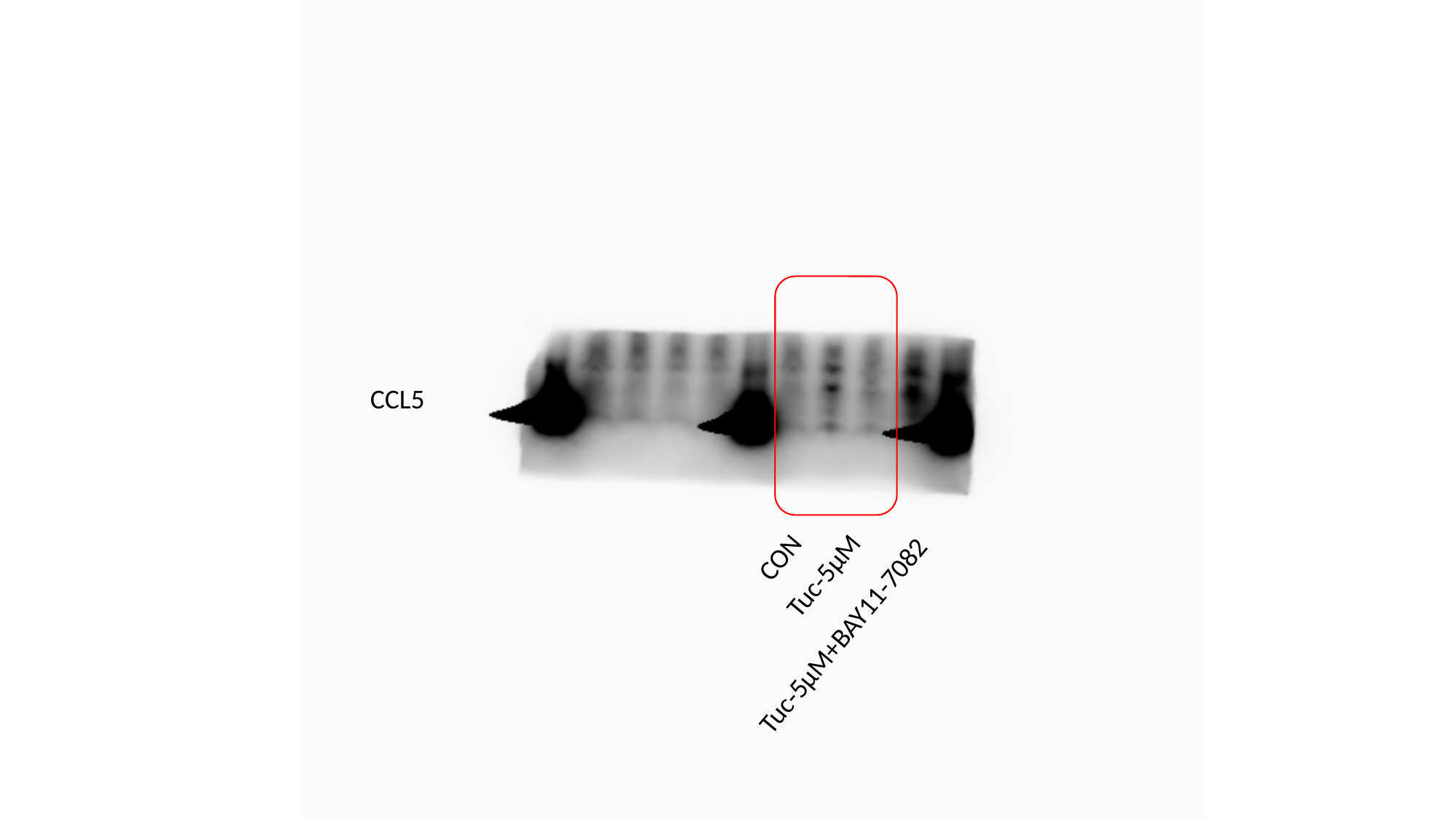

CCL5
CON
Tuc-5μM
Tuc-5μM+BAY11-7082

## Slide 7
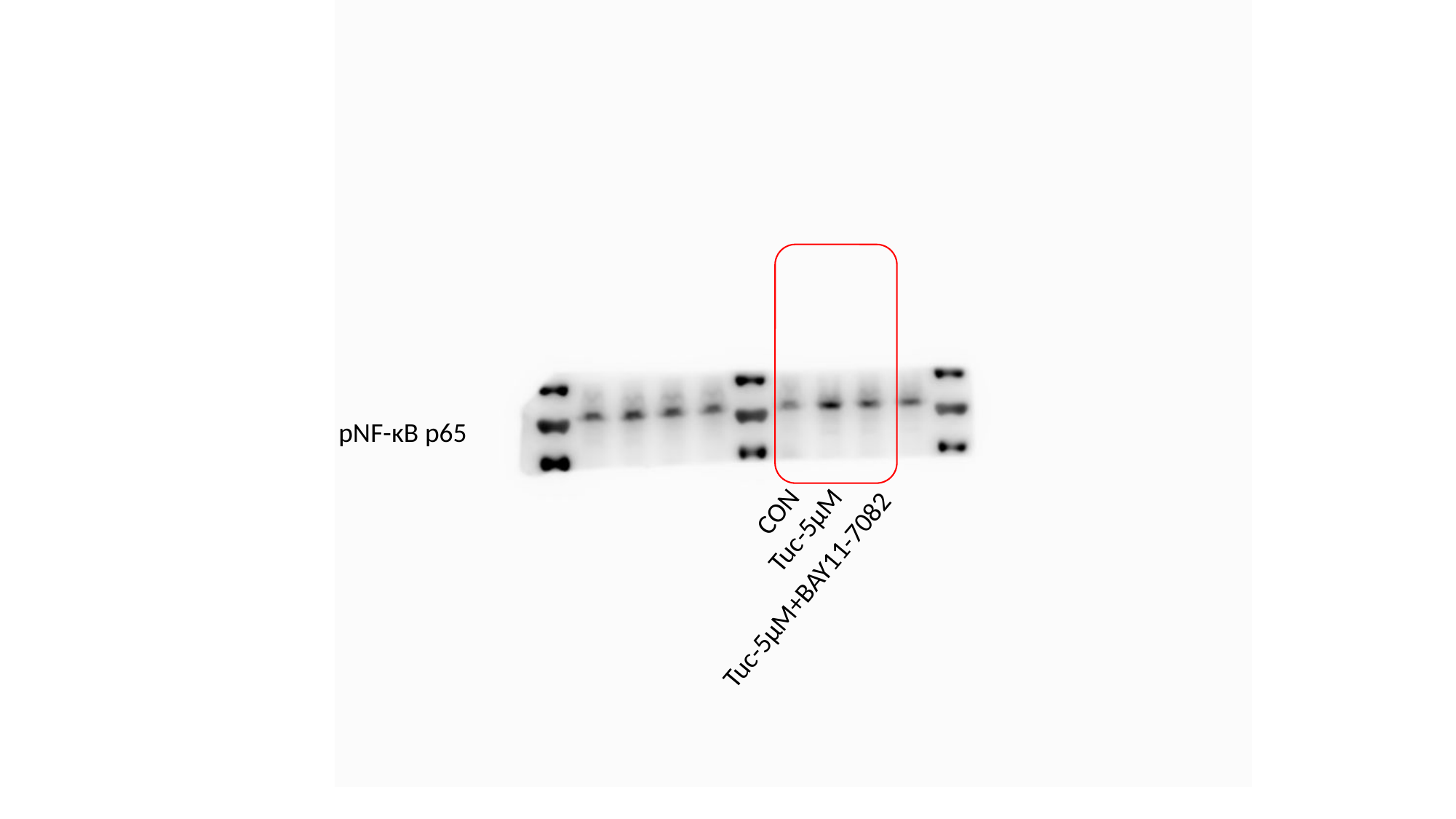

pNF-κB p65
CON
Tuc-5μM
Tuc-5μM+BAY11-7082

## Slide 8
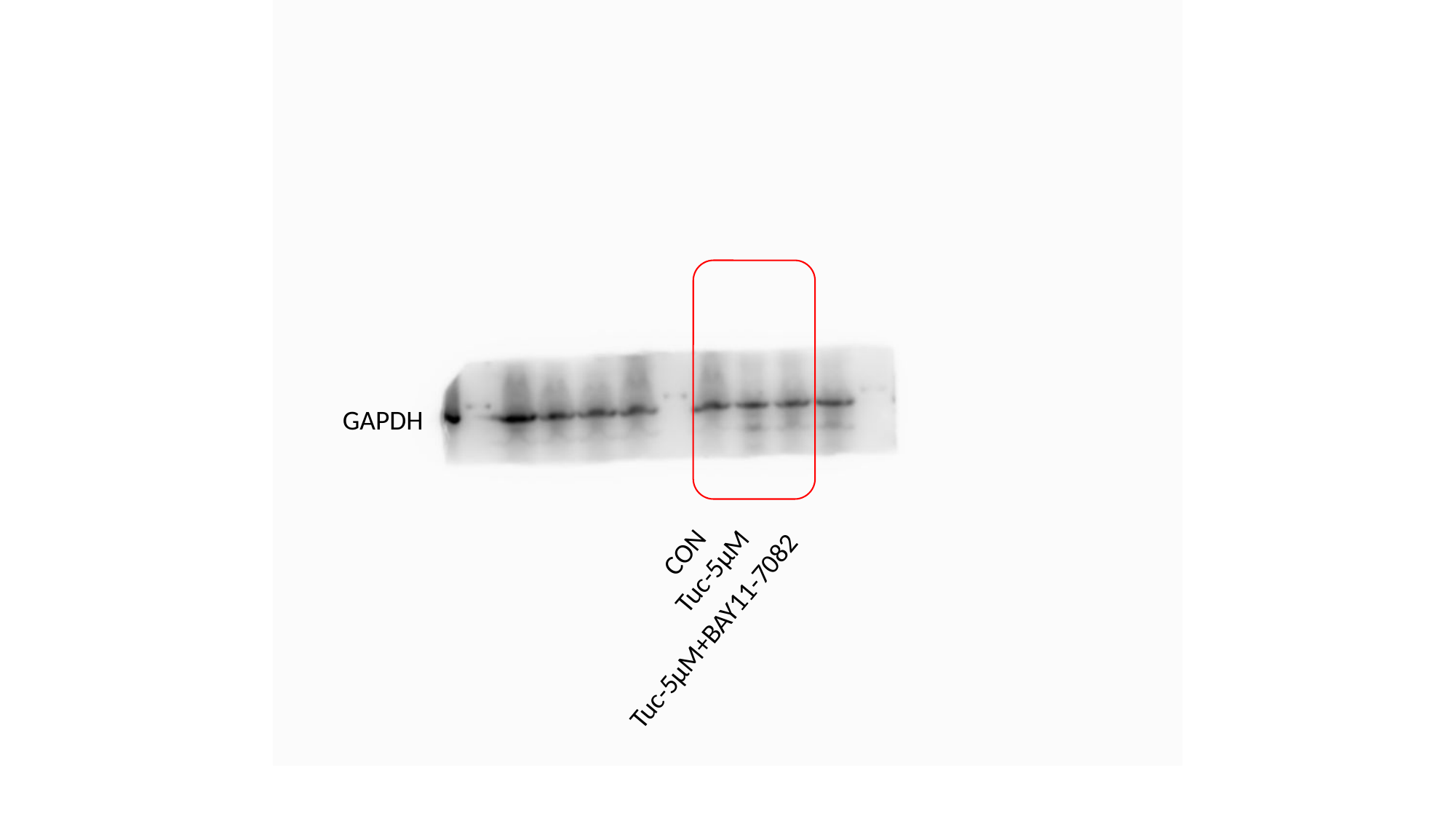

GAPDH
CON
Tuc-5μM
Tuc-5μM+BAY11-7082
